# IL-33 receptor ST2 regulates the cognitive impairments associated with experimental cerebral malaria

**DOI:** 10.1371/journal.ppat.1006322

**Published:** 2017-04-27

**Authors:** Flora Reverchon, Stéphane Mortaud, Maëliss Sivoyon, Isabelle Maillet, Anthony Laugeray, Jennifer Palomo, Céline Montécot, Améziane Herzine, Sandra Meme, William Meme, François Erard, Bernhard Ryffel, Arnaud Menuet, Valérie F. J. Quesniaux

**Affiliations:** 1 CNRS, UMR7355, Orleans, France; 2 Experimental and Molecular Immunology and Neurogenetics, University of Orleans, Orleans, France; 3 Division of Rheumatology, Departments of Internal Medicine Specialties and of Pathology-Immunology, University of Geneva School of Medicine, Geneva, Switzerland; 4 CNRS, Centre de Biophysique Moléculaire, Orléans, France; Albert Einstein College of Medicine, UNITED STATES

## Abstract

Cerebral malaria (CM) is associated with a high mortality rate and long-term neurocognitive impairment in survivors. The murine model of experimental cerebral malaria (ECM) induced by *Plasmodium berghei* ANKA (*Pb*A)-infection reproduces several of these features. We reported recently increased levels of IL-33 protein in brain undergoing ECM and the involvement of IL-33/ST2 pathway in ECM development. Here we show that *Pb*A-infection induced early short term and spatial memory defects, prior to blood brain barrier (BBB) disruption, in wild-type mice, while ST2-deficient mice did not develop cognitive defects. *Pb*A-induced neuroinflammation was reduced in ST2-deficient mice with low *Ifng*, *Tnfa*, *Il1b*, *Il6*, *CXCL9*, *CXCL10* and *Cd8a* expression, associated with an absence of neurogenesis defects in hippocampus. *Pb*A-infection triggered a dramatic increase of IL-33 expression by oligodendrocytes, through ST2 pathway. *In vitro*, IL-33/ST2 pathway induced microglia expression of IL-1β which in turn stimulated IL-33 expression by oligodendrocytes. These results highlight the IL-33/ST2 pathway ability to orchestrate microglia and oligodendrocytes responses at an early stage of *Pb*A-infection, with an amplification loop between IL-1β and IL-33, responsible for an exacerbated neuroinflammation context and associated neurological and cognitive defects.

## Author summary

The cerebral complication of malaria caused by *Plasmodium falciparum* infection, is associated with long-term neurological sequelae in survivors. The mechanisms involved in neurocognitive impairments during cerebral malaria development are still unknown. We reported recently the essential role of IL-33/ST2 pathway in experimental cerebral malaria (ECM) development. In this study we investigated the capacity of IL-33, highly expressed in oligodendrocytes, to promote ECM-associated neurological and cognitive damages. We found that IL-33/ST2 pathway through glial cells is involved in neurocognitive impairments, associated with exacerbated neuroinflammation, and altered neurogenesis. Interestingly, the implication of glial cells with a high level of IL-33 production in neurocognitive disorders, occurs at an early stage of ECM development, prior to blood brain barrier permeabilization. We propose the link between microglial IL-1β and oligodendrocytes IL-33 production in neurological symptoms associated with ECM.

## Introduction

Malaria is still one of the most devastating infectious diseases worldwide with 300–500 million cases each year and one million deaths every year (WHO). Cerebral malaria is a frequent cause of death from *Plasmodium falciparum* infection [1], with severe anemia, shock, lung vascular leakage, acute respiratory distress syndrome (ARDS) and neurological symptoms such as coma and seizures. In the late stage, the pathophysiology of cerebral malaria involves sequestration of erythrocytes, platelets and leukocytes in cerebral blood vessels leading to micro-vessel obstruction and excessive inflammation in the brain [1–3]. The increased immune response, involving CD8^+^ T cell and macrophage recruitment and the expression of pro-inflammatory cytokines, such as IFN-γ, TNF-α and Lymphotoxin (LT)-α, is central to the development of cerebral malaria, in both human and mice [4–9].

As in humans, increased levels of inflammatory cytokines and chemokines are present in the brain of *Plasmodium berghei* ANKA (*Pb*A) infected mice during experimental cerebral malaria (ECM) [10, 11]. We showed recently that IL-33 protein levels are increased in the brain undergoing ECM, and that mice deficient for IL-33 receptor ST2 display resistance to ECM after *Pb*A sporozoite or blood stage infection with a reduced ICAM expression and microvascular obstruction in the brain, and with decreased pathogenic T cell sequestration and LT-α induction [11]. At steady state, part of the immune cells present in the brain, including some CD4^+^ and CD8^+^ T cells, NKT cells and granulocytes, express ST2 receptor. But none of those ST2^+^ immune cells were clearly increased in the brain undergoing ECM [11], suggesting that IL-33/ST2 pathway from other cell types, such as in central nervous system (CNS) cells, may be involved.

Cerebral malaria is also associated with long-term neurocognitive impairment in 20% of infected children [12, 13]. Surviving patients have an increased risk of neurological and cognitive deficits, behavioral changes and seizures, making cerebral malaria a leading cause of childhood neurodisability in sub-Saharan Africa [14], and calling for the improvement of adjunctive therapies for reducing the cognitive sequelae. Thus, it is essential to better understand the link between *Plasmodium*-induced neuroinflammation and cognitive impairments to identify relevant targets. Here, we tested the hypothesis that IL-33, an alarmin with multiple functions which is highly expressed in the brain, may contribute to ECM-associated cognitive defects.

In mice, a link between ECM induced by *Pb*A and cognitive dysfunction was reported [15]. During ECM, brain damage occurs in regions known to be important for cognition, such as the fornix, cortex, and hippocampus [15]. In a C57BL/6 mouse model of *Pb*A-induced ECM, impairment in the visual memory at 1h and in object-recognition test of working memory were described on day 7 post-infection [15]. This cognitive dysfunction correlated with hemorrhages and inflammation, and with microglial activity associated with morphological changes throughout the brain of *Pb*A-infected mice [15].

Cognitive impairment in human cerebral malaria was associated with high cytokine levels in the cerebrospinal fluid (CSF) including IL-6, CXCL-8/IL-8, G-CSF, TNF-α and IL-1Ra, whereas a correlation between serum and CSF levels was only found for G-CSF [16]. IL-33 levels are increased in the plasma of infants with severe malaria, as compared with infection-free controls [17]. In mice, IL-33 was associated with some cerebral diseases, including *Toxoplasma gondii*-induced encephalitis [18], while it was also shown to attenuate experimental autoimmune encephalomyelitis (EAE) by suppressing IL-17 and IFN-γ production [18, 19]. Further, daily systemic exogenous administration of IL-33 induced ILC2s expansion, Th2 cytokines release, anti-inflammatory M2 macrophages polarization and Treg cells expansion that eventually suppressed the proinflammatory response and prevented ECM development [20]. Indeed, while IL-33 was originally described as an inducer of type 2 immune responses, its pleiotropic nature is now well documented, including in inducing type 1 responses, and exogenous systemic IL-33 treatment may either protect or exacerbate infections, depending on the infectious disease (for review [21]). Here we focused on the implication of the endogenous IL-33/ST2 pathway in ECM-associated CNS and cognitive defects. Among the different nervous system cell types, brain endothelial cells, astrocytes, and more recently oligodendrocytes have been described as effective sources of IL-33, both in brain and spinal cord [22–24]. IL-33 receptor ST2 was shown to be expressed, among others, by astrocytes, microglial cells, neurons and brain endothelial cells, presenting those cells as potential targets of IL-33 [22]. Further, an increase of ST2 receptor expression was shown on the glial cell surface after spinal cord injury [25].

Given the importance of glial cells, including astrocytes, microglia and oligodendrocytes, to maintain the blood brain barrier (BBB), support neuronal functions and interact with the immune system, we hypothesized that the IL-33/ST2 pathway, especially in glial cells, may be involved in the ECM-associated neurological and cognitive damages. To address this hypothesis, we used a combination of *in vivo* studies and *in vitro* glial cell cultures, in the presence or absence of IL-33/ST2 pathway. We provide evidence that the *Pb*A-induced up-regulation of IL-33 protein in hippocampus was associated with short term memory impairment, pro-inflammatory cytokine production by activated microglia and altered neurogenesis. Based on our results, we show that IL-33/ST2 pathway is central for *Pb*A-induced cognitive impairments and we propose a critical link between IL-1β produced by microglia and the response of oligodendrocytes with IL-33 production, involved in cognitive defects before appearance of ECM related symptoms.

## Results

### Cellular sources of IL-33 in the brain of *Pb*A-infected mice undergoing cerebral malaria

**Fig 1:**
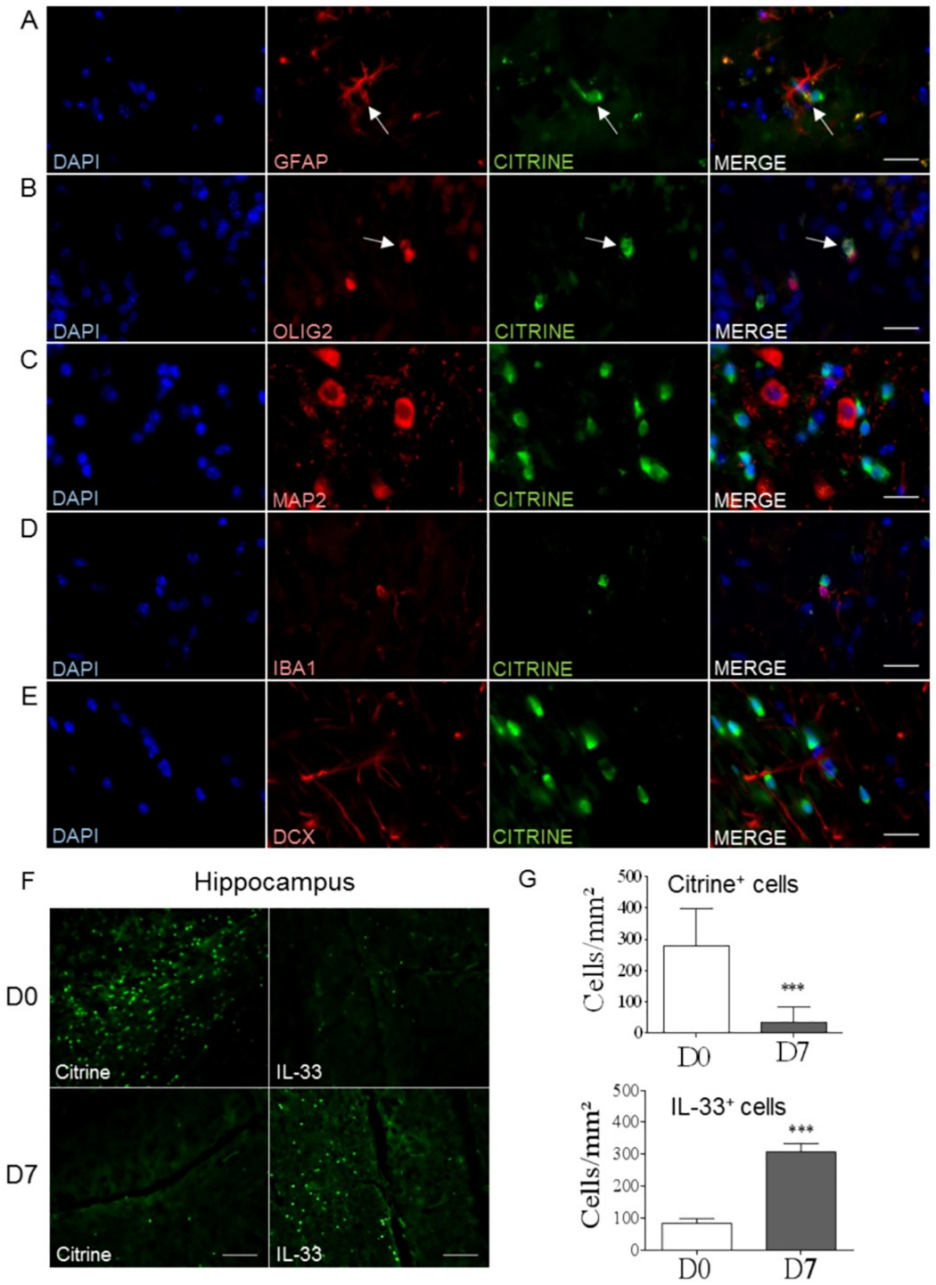
IL-33 expression by CNS resident cells and regulation after *Pb*A-infection. (A-E) To determine the cellular sources of IL-33 in CNS of naïve mice, we used IL-33/citrine reporter mice, with citrine (in green) as reporter gene for IL-33 expression. Immunofluorescence staining were realized on frontal cortex sections with markers for astrocytes (GFAP), oligodendrocytes (OLIG2), neurons (MAP2), microglia (IBA1) and neuroblasts (DCX) (in red). Scale bar 20μm. (F) Brain sections from naïve or 7-day-*Pb*A-infected IL-33/citrine reporter mice were analyzed to get representative images from hippocampus, with fluorescent citrine to visualize IL-33 promoter activity and with antibody immunofluorescence staining of IL-33 protein. Scale bar 100μm. (G) Cell counts of citrine^+^ cells and IL-33^+^ cells from previous images, F. These results are representative of 2 independent experiments and shown as mean ± SEM with n = 5. Mann Whitney test was applied (***p≤0.*001)*.

IL-33 is strongly expressed within the CNS [22], and we recently showed that the high steady-state levels of IL-33 protein found in naïve brain doubled during ECM, after blood stage or sporozoite *Pb*A-infection [11]. Since IL-33/ST2 has been implicated in *Pb*A-induced ECM, we first assessed the cellular source of IL-33 brain expression. Using IL-33/citrine reporter mice (IL-33^cit/+^; [26]) we confirmed the presence of IL-33 in the frontal cortex of naïve mice, that colocalized with markers of specific cellular populations (Fig 1). A colabelling of citrine was evident in cells expressing Glial fibrillary acidic protein (GFAP) and Oligodendrocyte transcription factor 2 (OLIG2), but not Microtubule-associated protein 2 (MAP2), Ionized calcium binding adaptor molecule 1 (IBA1) or Doublecortin (DCX) (Fig 1A–1E), indicating IL-33 expression in astrocytes and oligodendrocytes, but not in neurons, microglia or neuroblasts. IL-33 expression is regulated both at the transcriptional and translational level (for review, [27]; see also [23]). We reported earlier that the overall expression of *Il33* mRNA was not significantly altered in the brain of wild-type (WT) C57BL/6 mice during ECM after *Pb*A-infection [11]. Here, by analyzing specifically IL-33 expression in discrete CNS areas such as hippocampus, subventricular zone (SVZ) and frontal cortex, we showed a decrease of IL-33 promoter activity and an overexpression of IL-33 protein, with nuclear labeling [23], in the hippocampus (Fig 1F and 1G), the subventricular zone and the cerebellum (S1A and S1B Fig). Thus, we confirm not only that IL-33 is expressed in astrocytes and oligodendrocytes in the brain, but that this expression is increased in hippocampus 7 days post *Pb*A-infection.

### ST2 pathway is essential for the cognitive defects associated with ECM induced by *Pb*A-infection

**Fig 2:**
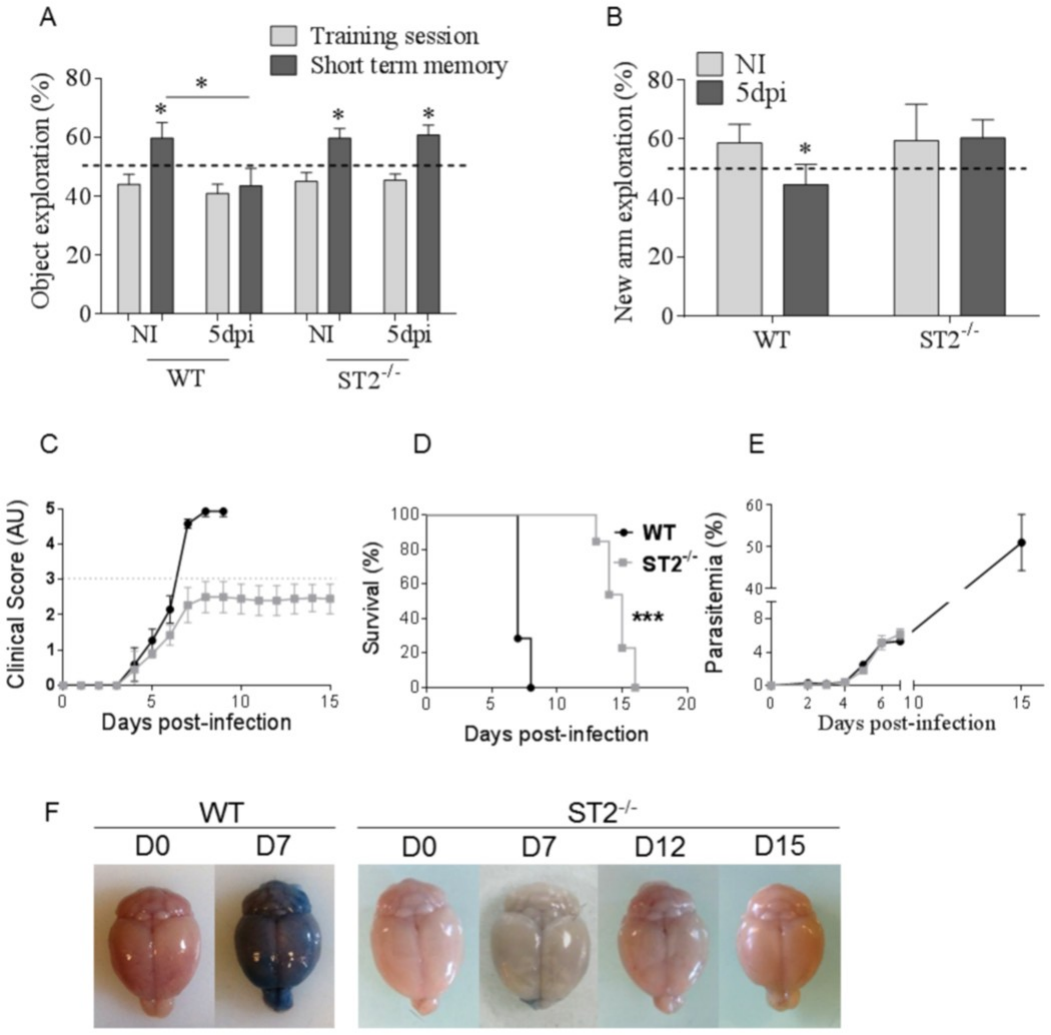
*Pb*A-induced impairment of cognition is prevented in the absence of IL-33/ST2 pathway. ST2^-/-^ and WT mice were infected with 10^5^ *Pb*A-parasitized red blood cells. (A) Novel object recognition (NOR) investigated short term memory impairment 5 days post-infection (dpi). These results are representative of 2 independent experiments and shown as mean ± SEM with n = 9 to 12 mice per group. NOR was compared to the respective training session (*on the bar). (B) Y maze was used to assess hippocampal memory 5 dpi. These results are shown as mean ± SEM with n = 10 to 15 mice for WT and n = 9 to 10 for ST2^-/-^ mice. One-way ANOVA followed by Bonferroni post-test (*p≤0.05). (C-E) Survival was followed (C), neurological signs were observed and translated into a clinical score, expressed in arbitrary units (AU) (D), and parasitemia was measured until day 7 (E). (F) Blood brain barrier leakage was assessed after Evan’s Blue injection on day 7 after *Pb*A-infection in WT and ST2^-/-^ mice (n = 4) and further on day 12 (n = 4) and 15 (n = 3) in surviving, ECM resistant ST2^-/-^ mice. Kruskal-Wallis was applied, followed by Dunn’s comparison test (***p≤0.*001)*.

We next assessed the potential implication of IL-33/ST2 pathway in the cognitive impairment associated with *Pb*A-infection and ECM development. Novel object recognition (NOR) test was chosen to investigate short term memory impairment associated with hippocampus dysfunction, after a training session with familiar objects. Short term memory was reduced 5 days after *Pb*A-infection in WT mice, while the cognition process remained unaltered in the absence of IL33/ST2 pathway (Fig 2A). We then confirmed these cognitive defects with a second memory test, namely the Y maze test, based on spontaneous exploration ability, used to study hippocampus function [28, 29]. After a training session with 2 arms, mice were tested with an additional, novel opened arm. Naïve WT and ST2^-/-^ mice typically spend most time in the novel opened arm. Five days post-*Pb*A infection WT mice presented a decreased exploration of the new arm, while infected ST2^-/-^ mice behaved like naïve mice and spent *ca* 60% time in the new arm (Fig 2B). In our two cognitive test conditions, no difference of locomotor activity was observed between the two mouse groups (S2A and S2B Fig). Furthermore we confirmed that ST2^-/-^ mice were protected from *Pb*A-infection-induced ECM as demonstrated by a survival beyond day 13, a slight delay in the apparition of neurological signs, and a reduced neurological score, while WT mice developed neurological symptoms and died from ECM within 7–8 days (Fig 2C and 2D). Parasitemia was similar in both groups until day 7 (Fig 2E), and continued to increase in ECM resistant ST2^-/-^ mice, which eventually died of hyperparasitemia (51% ±6 on day 15) and anemia, as reported [11], without blood brain barrier disruption (Fig 2F). Since long-term cognitive impairment occur in human cerebral malaria, murine models have been developed after *Pb*A-infection and treatment with chloroquine to investigate cognitive deficits up to 30–40 days after infection [30, 31]. Here, ECM resistant ST2-deficient mice were protected from ECM associated cognitive impairment from day 5 to up to day 11 post-infection (S3A Fig), after what their locomotion was impaired (S3B Fig), due to the severe anemia. Therefore, the short memory impairment seen on day 5 post-*Pb*A infection in WT mice was absent in ST2 deficient mice, indicating that the IL-33/ST2 pathway is involved in the cognitive defects seen at this early stage of ECM development.

### Drastic and late onset of ECM-induced BBB leakage and vascular damage on day 7 post-*Pb*A infection

**Fig 3:**
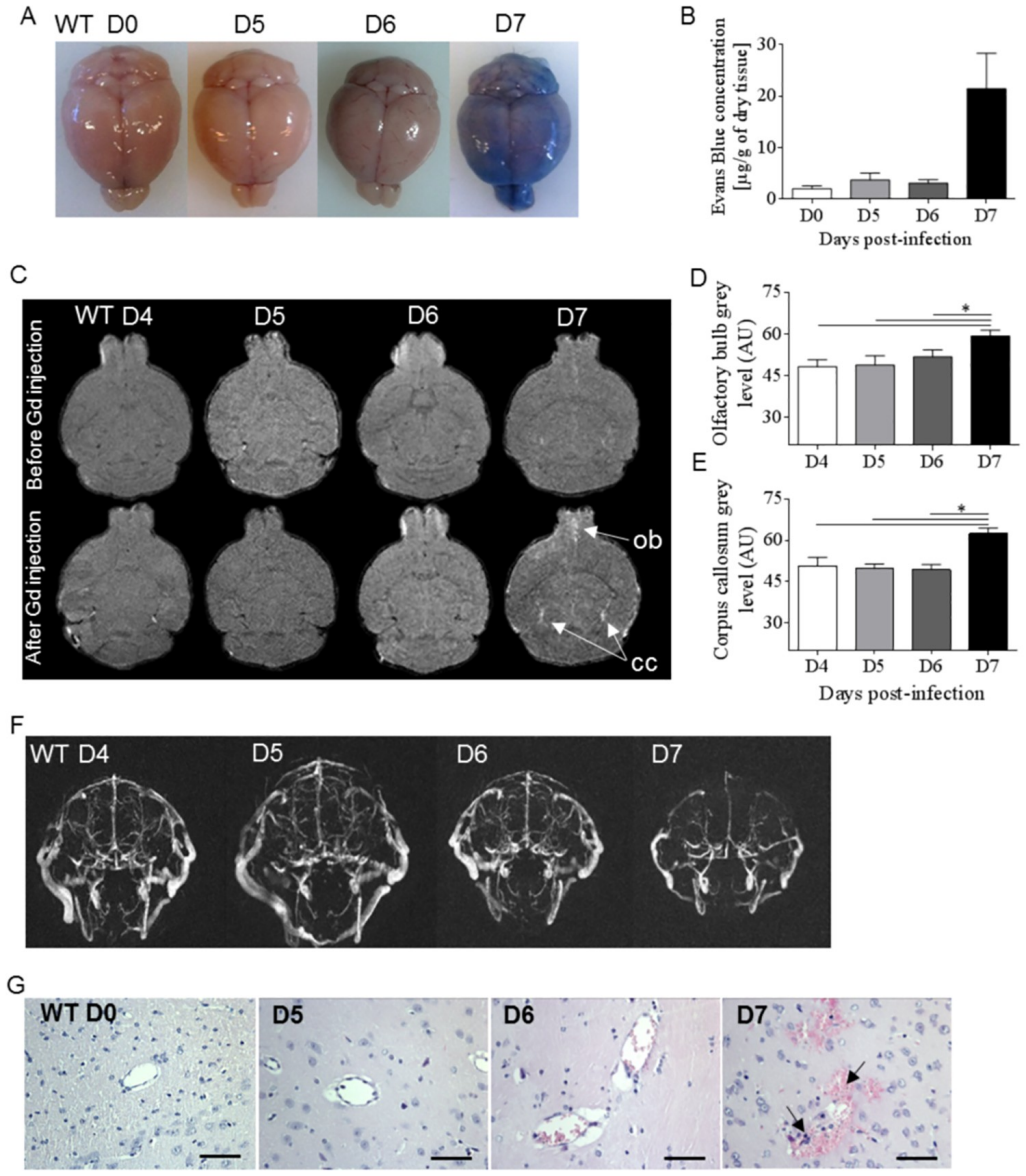
Kinetics of ECM-induced BBB leakage and vascular damage after *Pb*A-infection. (A-B) Blood brain barrier permeabilization was investigated in WT mice with Evan’s Blue injection on day 5, 6 and 7 after *Pb*A-infection, and compared to naïve mice. Typical images (A) and bar graph of Evan’s blue leakage (B) expressed as μg of blue per g of dry tissue (n = 3 per day). (C-F) Brain imaging using MRI and MRA analysis in *Pb*A-infected WT mice at early time points on day 4, 5, 6 and during late ECM on day 7 post-infection. Brain longitudinal MRI images of *Pb*A-infected WT mice before and after intravenous injection of Gadolinium (Gd) with distinct anomalies in the olfactory bulb (ob) and the corpus callosum (cc) are indicated by arrows (C). Bar graphs of olfactory bulb (D) and corpus callosum (E) mean grey level intensity from MRI images after Gd injection are shown (n = 5 per group). Representative images of vascular blood flow perturbation analyzed by MRA of *Pb*A-infected WT mice 4 to 7 days post-infection (F). MR images are representative of 5 mice per day. (G) Brain histology assessed on day 5, 6 and 7 after *Pb*A-infection in WT mice. Arrows indicate red blood cell / leucocyte sequestration and hemorrhages (hematoxylin-eosine staining; scale bar 50μm). Representative images of 2 independent experiments. Kruskal-Wallis was applied, followed by Dunn’s comparison test (*p≤0.*05)*.

To evaluate whether the cognitive defects observed in WT mice on day 5 post-*Pb*A infection were correlated with BBB leakage, Evans-Blue was i.v. injected to mice on day 5 to 7 after infection and compared to naïve mice (Fig 3A and 3B). We document an abrupt onset of BBB disruption on day 7 post-infection, corresponding to full-blown ECM in *Pb*A-infected WT mice, with no blue extravasation into brain tissue up to day 6 post-infection. We further characterized by magnetic resonance imaging (MRI) and angiography (MRA) the neuropathogenesis in WT mice after 4, 5, 6 and 7 days post-*Pb*A infection. These noninvasive tools are used for neurological disease investigation during cerebral malaria in human patients [32–34] but also in murine ECM models, where they allow a semi-quantitative analysis of swelling/edema, focal ischemia, morphological changes and vascular blood flow [35], [36]. Here, the cerebral endothelium lesions were further detected by MRI in the presence of Gadolinium (Gd) and revealed an increase in signal intensity in olfactory bulbs (ob) and corpus callosum (cc), which appeared only on day 7, in ECM undergoing WT mice (Fig 3C–3E). In line with these results, we show by MRA a dramatic reduction of vascular blood flow 7 days post-*Pb*A infection in WT mice (Fig 3F), associated with micro-hemorrhages and leucocyte sequestration in brain microvessels on day 7 after infection (Fig 3G).

Thus, there was no cerebral vascular leakage or microcirculation obstruction before day 7 post-*Pb*A infection in WT mice, indicating that the *Pb*A-induced early cognitive defects documented on day 5 occurred prior to BBB damage.

### ECM-associated neurogenesis defect is absent in ST2 deficient mice

**Fig 4:**
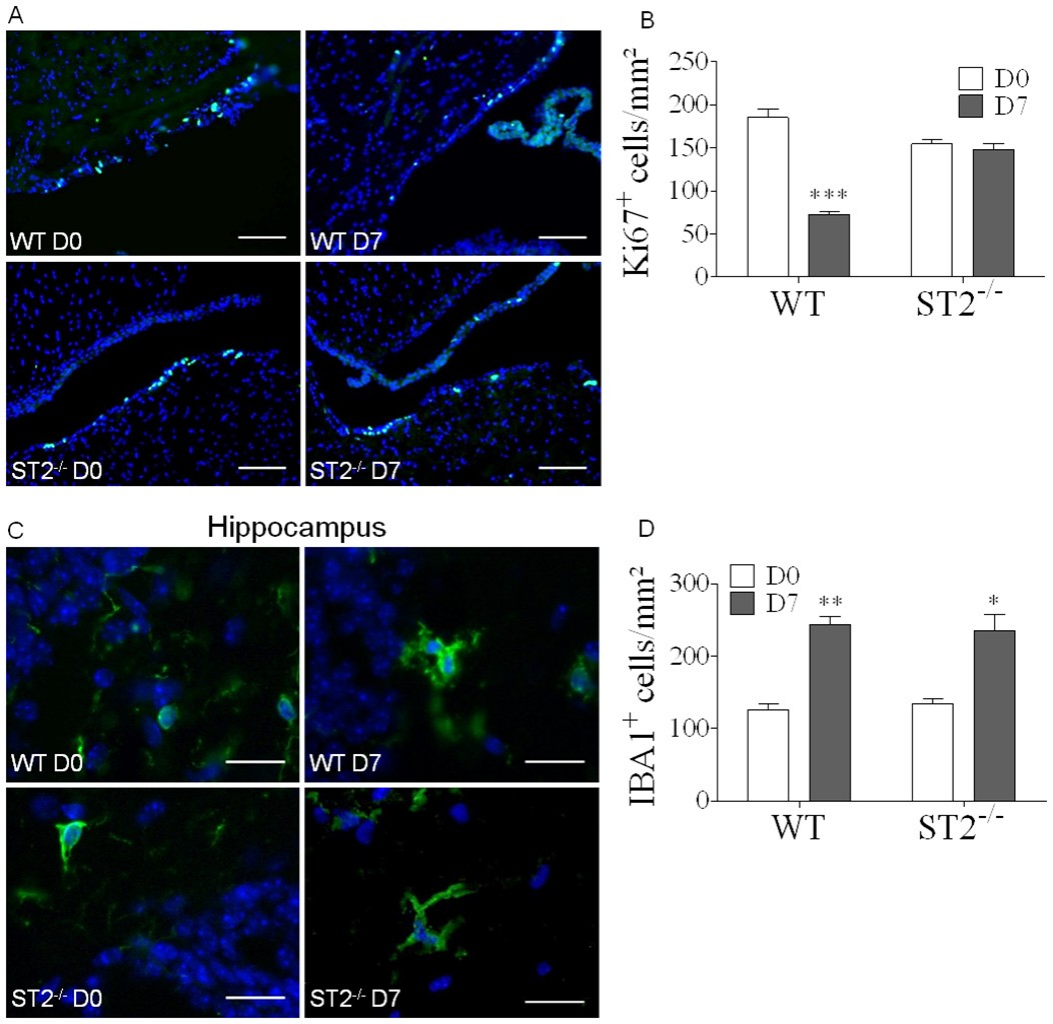
Preserved neuron proliferation after *Pb*A-infection in the absence of IL-33/ST2 pathway. (A) Immunofluorescent staining of cell proliferation marker Ki67, showing neurogenesis in the ventricle wall in WT and ST2^-/-^ mice 7 days post-*Pb*A infection. Scale bar 100μm. (B) Cell counts of Ki67^+^ cells in ventricle wall sections from WT and ST2^-/-^ infected or naïve mice. These results are expressed as mean ± SEM, n = 4 to 5 mice in 2 independent experiments with 5 fields analyzed per mouse. (C) Immunofluorescent staining of microglia marker IBA1, on brain sections, showing microglia morphology in hippocampus from WT and ST2^-/-^ mice, 7 days post-*Pb*A-infection. Scale bar 20μm. (D) Cell counts of IBA1^+^ cells from C, to compare microglia proliferation in WT and ST2^-/-^ brain, 7 days post-*Pb*A infection. These results are expressed as mean ± SEM, n = 3 mice per group in 2 independent experiments with 5 fields analyzed per mouse. Kruskal-Wallis was applied, followed by Dunn’s comparison test (*p≤0.*05*, **p≤0.*01*, ***p≤0.*001)*.

Neurogenesis defined as the complex process of functional neuron formation from neural stem cells and progenitor cells, is essential for cognition. A disturbed neurogenesis was described in adult WT mice by a single episode of mild malaria after *P*. *chabaudi adami* infection (37). Here, Ki67 immunostaining of neuron proliferation in the ventricle wall, showed a clear reduction of proliferating cells 7 days after *Pb*A-infection in WT mice, while there was no reduction in infected ST2^-/-^ mice (Fig 4A and 4B), suggesting that neurogenesis was preserved after *Pb*A-infection in the absence of IL-33/ST2 pathway.

Given that neurogenesis can be influenced by microglia [37], we next explored the status of microglia activation in the absence of ST2 in *Pb*A-infected mice. Further, changes in microglia architecture may be associated with neuronal deficits [38]. We assessed microglia morphology and proliferation in the hippocampus 7 days post *Pb*A-infection. Immunofluorescent staining revealed a morphology typical of activated microglia with hypertrophied cell body and thick cytoplasmic extension, together with an increase in IBA1^+^ cells in the hippocampus (Fig 4C and 4D) and cerebellum (S4A and S4B Fig) of *Pb*A-infected WT and ST2^-/-^ mice. Thus microglia activation and proliferation 7 days post-infection was independent of the IL-33/ST2 pathway.

### Glial cell inflammatory response after *Pb*A-infection is reduced in hippocampus in the absence of IL-33/ST2 pathway

**Fig 5:**
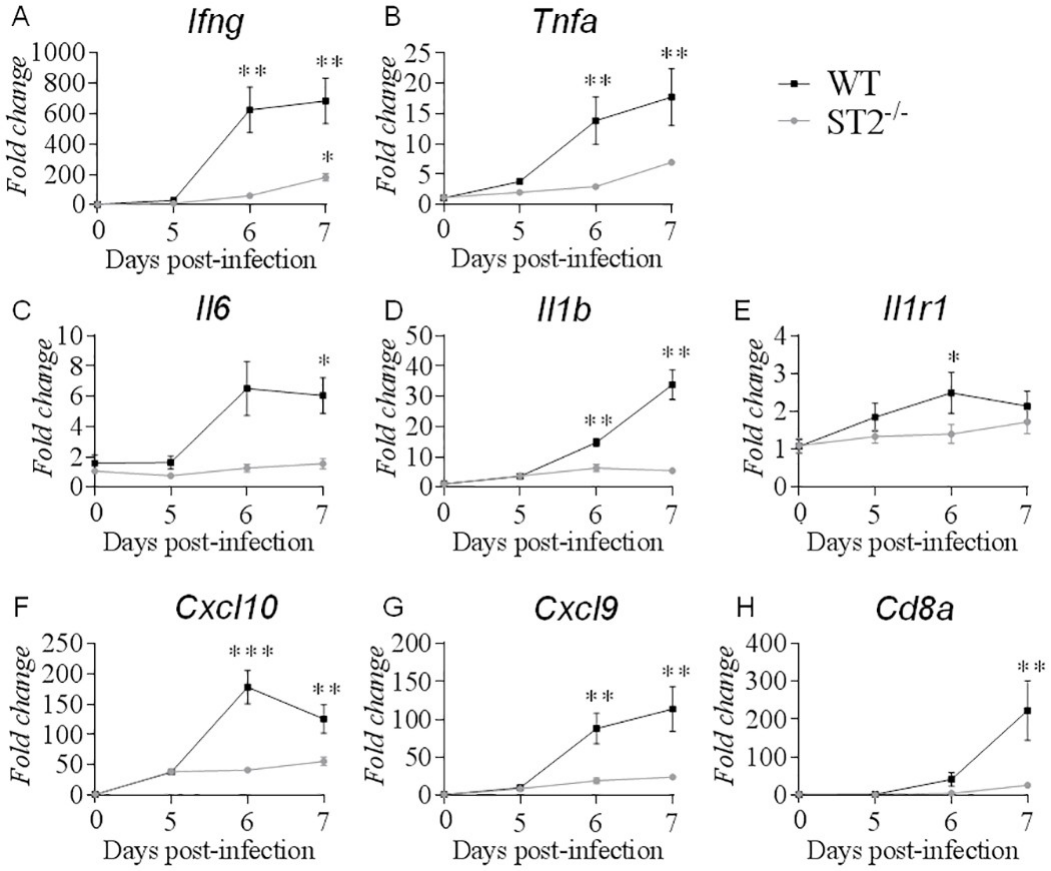
Inflammatory response in hippocampus after *Pb*A-infection is reduced in the absence of IL-33/ST2 pathway. Inflammation context in hippocampus was investigated in naïve and *Pb*A-infected WT or ST2^-/-^ mice on day 5, 6 and 7 after infection. (A-H) *Ifng*, *Tnfa*, *Il6*, *Il1b*, *Il1r1*, *Cxcl10*, *Cxcl9* and *Cd8a* mRNA expression in hippocampus were quantified by real-time quantitative RT-PCR. Expression of *18s* housekeeping gene was used for normalization. Results expressed as fold change relative to uninfected mice are mean ± SEM, n = 5 mice per group per day, representative of 3 independent experiments. Statistical analysis was done using Kruskal-Wallis test followed by Dunn’s comparison test (*p≤0.*05*, **p≤0.*01*, ***p≤0.*001)*.

CNS recruitment and activation of CD8^+^ effector T cells is essential for ECM pathogenesis [7, 8, 39, 40]. We reported previously an impaired sequestration of activated effector T cells in the brain of *Pb*A-infected mice on day 7 in the absence of IL-33/ST2 functional pathway [11]. The expression of T cell specific transcripts including *Cd8a* and *Ifng*, was strongly up-regulated in the brain 7 days post-*Pb*A-infection in WT mice, as compared to naïve control mice [11]. However, the whole brain overexpression of *Ifng* and *Cd8a* was similar or lower in *Pb*A-infected ST2^-/-^ mice, as compared to infected WT mice [11]. To further address glial cell function in the absence of IL-33/ST2 pathway, we next analyzed the kinetics of cytokine regulation in the hippocampus, an area involved in memorial processes, during early stage *Pb*A-infection (Fig 5). We document a sharp increase in the inflammatory response in terms of *Ifng*, *Tnfa*, *Il6*, *Il1b*, *Il1r1*, *Cxcl9* and *Cxcl10* expression already on day 6 post-infection in WT mice, and of *Cd8a* on day 7, which were essentially absent in ST2^-/-^ mice on day 5 to 7 after *Pb*A-infection (Fig 5A–5H). Similar results were obtained in frontal cortex for *Ifng*, *Tnfa*, *Il1b* and *Cd8a* expression, although it was more variable (S5A–S5D Fig). Therefore, the glial cell inflammatory response observed in hippocampus after *Pb*A-infection requires the presence of a functional IL-33/ST2 pathway.

### Increased IL-33 expression by oligodendrocytes in hippocampus upon *Pb*A-infection

**Fig 6:**
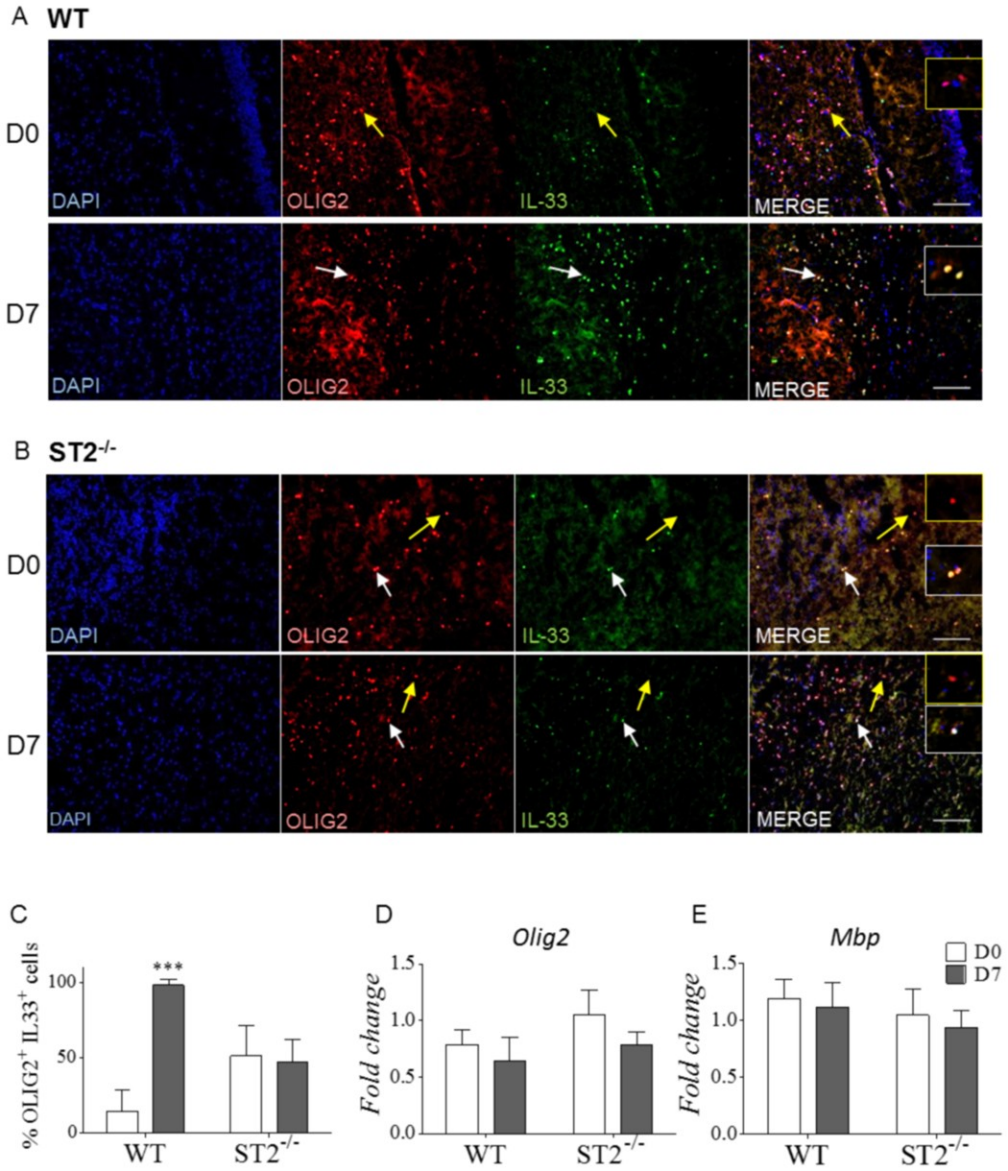
Increased IL-33 expression by oligodendrocytes in hippocampus upon *Pb*A-infection. (A-B) Immunofluorescence staining of IL-33 and oligodendrocytes marker OLIG2 on hippocampus sections of naïve or *Pb*A-infected WT or ST2^-/-^ mice 7 days post-infection. White arrows indicate colocalization (white insert) and yellow arrows an absence of colocalization (yellow insert). Scale bar 100μm. (C) Cell counts of double positive OLIG2^+^IL-33^+^ cells from above. These results are expressed as mean ± SEM, n = 3 mice, representative of 2 independent experiments with 5 fields analyzed per mouse. (D-E) *Olig2* and *Mbp* mRNA expressions in hippocampus from naïve or *Pb*A-infected WT or ST2^-/-^ mice on day 7 after infection were quantified by real-time quantitative RT-PCR. Expression of *18s* housekeeping gene was used for normalization. Results expressed as fold change relative to uninfected mice are mean ± SEM, n = 5 mice per group per day, representative of 3 independent experiments. Statistical analysis was done using Kruskal-Wallis test followed by Dunn’s comparison test (***p≤0.*001)*.

To better define the role of glial cells in ECM development, we further investigated oligodendrocytes, recently recognized as CNS immunomodulatory cells, rather than “defenseless victims” of brain inflammation caused by microglia activation [41], since oligodendrocytes express a wide range of immunomodulatory molecules (reviewed in [42]). Immunofluorescence staining was performed on brain of WT and ST2^-/-^ infected mice, and compared to naïve mice, to evaluate IL-33 production by oligodendrocyte after *Pb*A-infection (Fig 6A and 6B). The proportion of hippocampal OLIG2^+^ IL-33^+^ cells was sharply increased on day 7 post *Pb*A-infection in WT mice, but not in ST2^-/-^ infected mice (Fig 6C). Furthermore, *Olig2* (Oligodendrocyte transcription factor 2) and *Mbp* (Myelin basic protein) mRNA expression were maintained in hippocampus in WT and ST2^-/-^ infected mice (Fig 6D and 6E). Thus, *Pb*A-infection induced an inflammatory response in the hippocampus, involving a strong IL-33 expression by oligodendrocytes, and this response was dependent on a functional IL-33/ST2 pathway.

### IL-33/ST2 pathway is sufficient to trigger IL-1β production by microglia *in vitro*

We further investigated glial cell interactions through their ability to respond to and to produce cytokines in glial cell cultures prepared from newborn mouse cortical tissues. We first confirmed by immunofluorescence staining that IL-33 was produced by GFAP^+^ astrocytes and OLIG2^+^ oligodendrocytes, while by contrast, IL-1β was produced only by CD11b^+^ microglia, in response to a pro-inflammatory LPS stimulus (S6A and S6B Fig).

**Fig 7:**
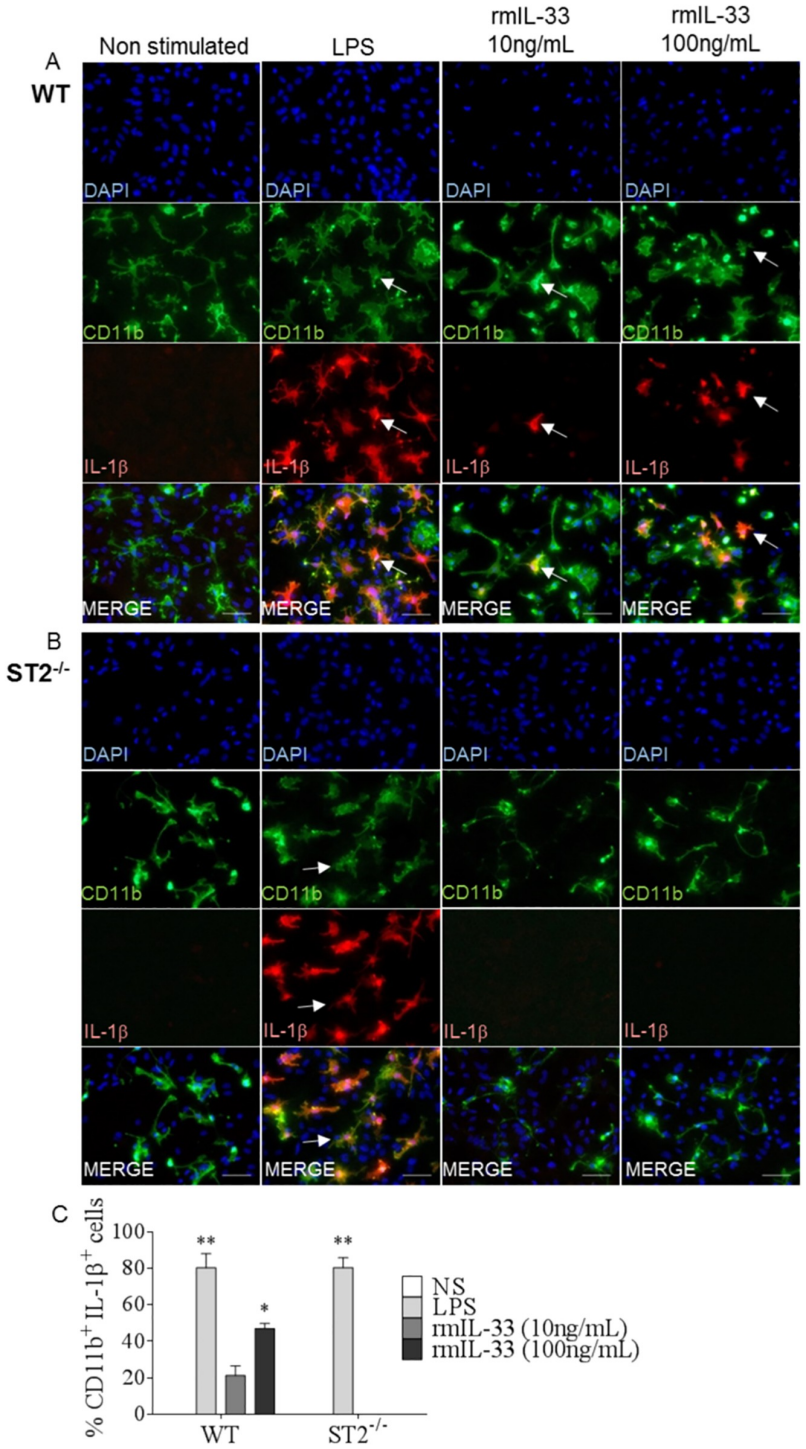
IL-33/ST2 pathway induces IL-1β production by microglia *in vitro*. (A-B) Mixed glial cell cultures from newborn WT (A) and ST2^-/-^ (B) mice were stimulated with rmIL-33 (10 or 100ng/mL) or LPS (10μg/mL), as a positive control for 24 hrs, or unstimulated, then fixed and used for immunofluorescence staining of IL-1β with microglia marker CD11b. Arrows indicate colocalization. These images are representative of 3 independent experiments. Scale bar 100μm. (C) Cell percentages of CD11b^+^ IL-1β^+^ cells from A and B. These results are expressed as mean ± SEM, n = 3 mice per group in 2 independent experiments with 5 fields analyzed per slide. Kruskal-Wallis was applied, followed by Dunn’s comparison test (*p≤0.*05*, **p≤0.*01)*.

In EAE, the overexpression of IL-33 by endothelial cells and astrocytes stimulated microglia induced IL-1β [22]. To further address the interactions of IL-1β and IL-33/ST2 pathway within glial cell communication pathways, we first confirmed that IL-33 induced IL-1β production by microglia. Indeed, in mixed glial cells cultures, rmIL-33 (10 or 100ng/mL) stimulation for 24 hrs induced morphological changes typical of activated microglia, together with IL-1β expression, LPS was used as positive control (Fig 7A). Immunofluorescence staining with colocalization of microglia marker CD11b and IL-1β demonstrates the cell-specificity of IL-1β production by microglia, as shown above after LPS stimulation (S6B Fig). In addition, IL-33 induced IL-1β production by microglia was dependent on the presence of the ST2 pathway (Fig 7B) since no IL-1β was detected in ST2^-/-^ cultures stimulated with rmIL-33 (Fig 7C). Interestingly, the trend towards increased IL-1β protein expression observed in the hippocampus on day 5 to 7 post*Pb*A infection in WT mice was not present in ST2 deficient mice (S7B Fig). Thus, IL-33 induces IL-1β production by microglia through ST2 receptor pathway.

### IL-1β induces IL-33 production by oligodendrocytes

**Fig 8:**
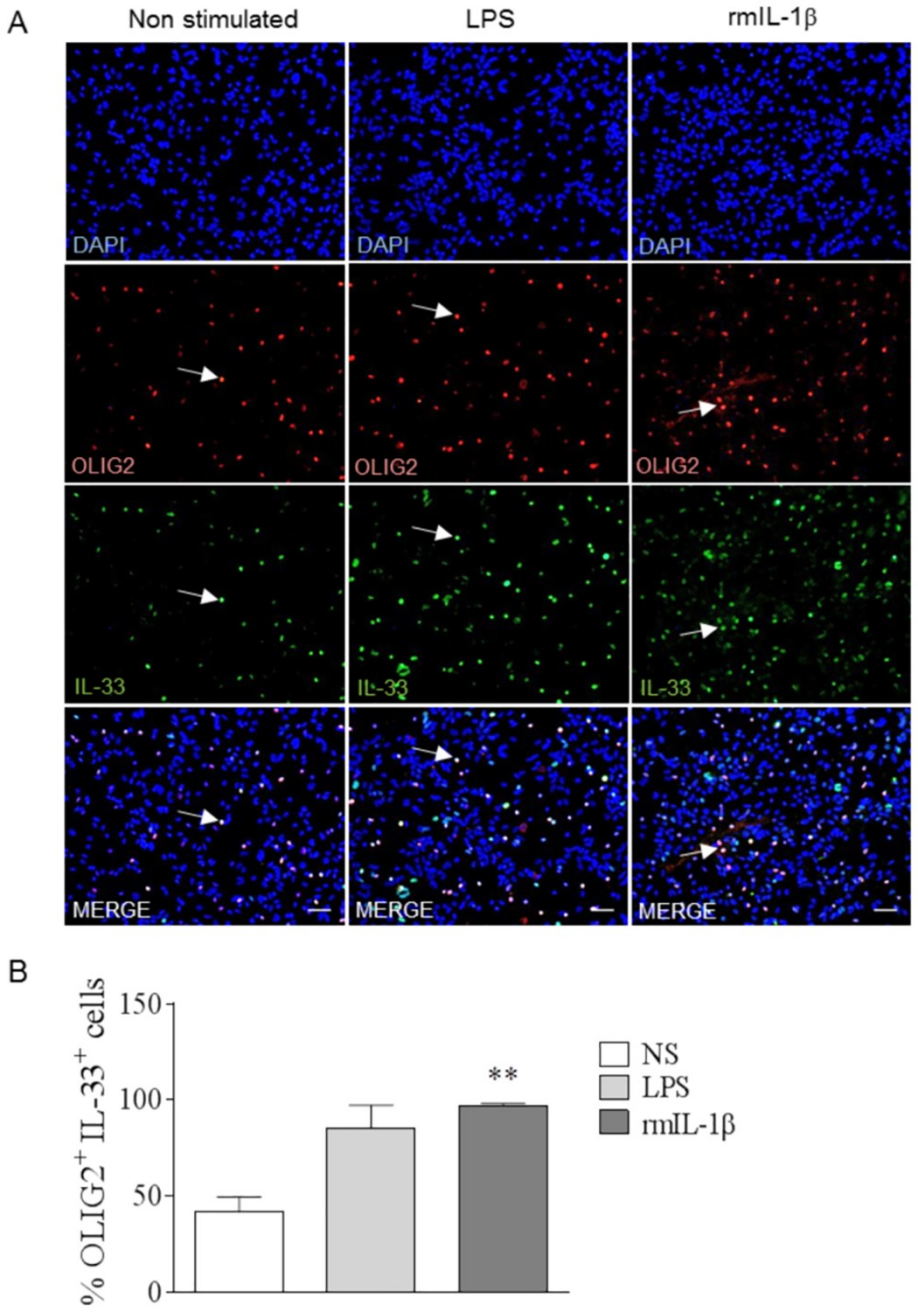
IL-1β induces IL-33 production by oligodendrocytes. (A) WT mixed glial cell cultures from newborn mice were stimulated with IL-1β (30ng/mL) or LPS (10μg/mL), as a positive control, for 24 hrs, then fixed and immunostained for OLIG2 and IL-33. These images are representative of 2 independent experiments. Arrows indicate colocalization of IL-33 with OLIG2. Scale bar 100μm. (B) Percentage of OLIG2^+^ cells expressing IL-33 from A. These results are expressed as mean ± SEM, n = 3 mice per group in 2 independent experiments with 5 fields analyzed per slide. Kruskal-Wallis was applied, followed by Dunn’s comparison test (**p≤0.01).

Microglia, considered as the classical innate immune cells of the CNS, may impact oligodendrocyte function (reviewed in [42]). Indeed, a recent study reported oligodendrocyte damage after IL-1β stimulation *in vitro* [43]. We thus investigated the possibility that, in turn, IL-1β produced by microglia could activate oligodendrocyte to induce IL-33 release, in line with our *in vivo* data. Mixed glial cell cultures were stimulated with rmIL-1β (30 ng/mL) for 24 hrs and immunofluorescence staining revealed a colocalization of oligodendrocyte nuclear marker OLIG2 and IL-33 expression (Fig 8A). Interestingly most OLIG2^+^ cells also stained positive for IL-33 after rmIL-1β stimulation (Fig 8B). Thus, IL-1β produced by microglia can induce IL-33 expression by oligodendrocytes.

## Discussion

Long-term neurocognitive impairments have been reported in one third of cerebral malaria survivors [12, 13, 44], calling for a better understanding of the connections between *Plasmodium*-induced neuroinflammation and cognitive defects. IL-33 is a potent immunomodulatory cytokine present in the CNS playing an essential role during neuroinflammatory processes [45]. In this study, we hypothesized that IL-33 may contribute to the cognitive defects associated with experimental cerebral malaria. Indeed, we document exacerbated IL-33 expression by astrocytes and oligodendrocytes after *Pb*A-infection in WT mice. Furthermore, we show early cognitive impairment on day 5 which depended on the IL-33/ST2 pathway and occurred prior to leucocyte sequestration in microvessels and BBB leakage induced by *Pb*A-infection on day 7.

While microglia activation was not affected by ST2 deficiency, the inhibition of neurogenesis and the production of inflammatory mediators in the hippocampus after *Pb*A-infection depended upon IL-33/ST2 pathway. In response to inflammatory stimuli, including IL-33 through ST2 pathway, microglial cells released IL-1β which in turn activated oligodendrocytes to produce high levels of IL-33, documenting a CNS endogenous IL-33 inflammatory loop that may contribute to *Pb*A induced cognitive disorders.

In view of these results it may seem paradoxical that administration of IL-33 prevented ECM development [19]. However, exogenous IL-33 exerts pleiotropic effects, beneficial or harmful, depending on the context of the disease [20], through a series of downstream direct and indirect effects [26]. Indeed, the effect of IL-33 given systemically on ECM development was indirect, mediated by systemic ILC2, Th2, M2 and Treg cell responses [19]. Understanding the biology of intracellular versus extracellular IL-33 will be required to differentiate the effects of homeostatic IL-33 acting locally on gene transcription or as an alarmin to organize immune responses at the site of barrier breaching, from IL-33 systemic effects as a cytokine [46]. Indeed, endogenous IL-33 is also reported to act within the cell, translocating to the nucleus, where it associates with chromatin and regulates gene expression through different mechanisms, and is stored as an alarmin to be released from dying cells. During apoptosis, IL-33 is cleaved and inactivated by apoptotic caspase-3 and caspase-7, while full-length IL-33 is released during necrosis. Biologically active full-length IL-33 is processed by different proteases yielding a shorter and more active form of 110–266 aa in mice, whereas disulfide bridge formation by oxidization inactivates IL-33 binding to its receptor. Recombinant IL-33 used in experimental pharmacological interventions is typically of the Ser109-Ile266 sequence, lacking the N-terminal non-classical nuclear localization sequence, and will thus mimic only few of IL-33 multifold activities.

IL-33 overexpression in CNS and spinal cord [47], including in astrocytes [48] and oligodendrocytes [23], has been observed in several neurological disorders, but its precise regulation remains unclear. Here, we show a decrease of *Il33* transcription and an upregulation of IL-33 protein in the hippocampus, the SVZ and the frontal cortex, 7 days post-*Pb*A infection in WT mice. These results suggest that astrocytes and oligodendrocytes release high levels of IL-33 protein from high basal levels of *Il33* mRNA, as reported for astrocytes and endothelial cells [22]. Pre-formed *Il33* transcripts allow the rapid translation of IL-33 protein in response to several stimuli without induction of new transcripts, as reported in spinal cord injury experiment [23].

In the CNS, astrocytes and microglia express ST2 and its co-receptor IL-1 accessory protein (IL-1RAcP), suggesting that both cell types may be primary targets for IL-33 [22] causing pro-inflammatory cytokine release [45]. Moreover, IL-33 induces microglia activation / proliferation that is a hallmark of neurological disorders such as CNS inflammation [22, 23].

ECM murine models have been questioned, but common features with human cerebral malaria include cytokine production in the brain, perturbations in blood-brain barrier integrity, iRBC sequestration to the cerebral microvasculature, vascular obstruction, either by iRBC, leukocytes, or both, microvascular damage, and persistent cognitive impairment [3, 9, 49, 50]. Indeed, neurological damage occurring during ECM development is in line with inflammatory conditions [51]. Our kinetics studies document an early cognitive defect in WT mice, which was absent in ST2^-/-^ mice, 5 days post-*Pb*A infection, at a time when there are no ECM-related neurological symptoms like ataxia or coma, no brain blood flow or BBB defect, before sequestration of inflammatory and CD8^+^ pathogenic T cells. ECM symptoms and brain vascular leakage have recently been associated with brain cell death and *Pb*A specific CD8^+^ T cells recruitment [52]. Thus, the early onset of *Pb*A-induced neurological disorders is independent of the BBB rupture. Immune cell sequestration during ECM development being concomitant with microvascular obstruction and BBB dysfunction on day 7, this suggested an involvement of non-immune cells, including CNS cells, in the inflammatory condition leading to *Pb*A-induced cognitive impairment.

Interestingly, *Pb*A-infection induced microglia activation and proliferation, even in the absence of functional IL-33/ST2 pathway. Indeed, microglia cells, considered as macrophage-like cells in the CNS can be stimulated by numerous immunomodulatory molecules [53] to produce high levels of cytokines, playing a prominent role in neuroinflammation and neuroprotection [22]. This suggests that other mechanisms, downstream of microglia activation, and likely IL-33/ST2 pathway dependent, are required to cause cognitive defects and ECM development after *Pb*A-infection.

Microglial activation and release of inflammatory cytokines such as IL-6, IFN-γ and TNF-α can affect the differentiation and proliferation of neuroblasts in SVZ, as shown in a murine model of Japanese encephalitis [54]. A recent study confirmed that neurogenesis could be affected by activated microglia [37], which in turn might impact the cognitive processes. Here, the significant reduction of neuroblast proliferation in *Pb*A-infected WT mice was prevented in the absence of IL-33/ST2 pathway, which, in line with the absence of cognitive defect in these mice, confirms a causality link between neurogenesis and cognitive defects.

Although microglia was activated in ST2 deficient mice after *Pb*A-infection, the absence of ST2 receptor impacted the production of pro-inflammatory molecules in hippocampus after *Pb*A-infection. Indeed, *Cxcl9* and *Cxcl10*, chemokines responsible for the recruitment of activated CD8^+^ T cells to the brain, and *Il1b*, a crucial molecule in inflammation, were drastically reduced in the absence of ST2 pathway, as compared to WT infected mice. These results suggest that IL-33/ST2 pathway affects in part microglia function and consequently explained normal neurogenesis in ST2^-/-^ infected mice. Moreover, in the absence of BBB rupture, an increased production of inflammatory cytokines in CNS, reported after *P*. *chabaudi adami* infection, was associated with inhibition of hippocampal neurogenesis, leading to cognitive defects [37]. Here, our results indicate for the first time an early, crucial role of IL-33/ST2 pathway in the development of neuroinflammation affecting neurogenesis during ECM development.

It has been published that IL-33 is an activator of microglia with IL-1β release leading to neuroinflammation [22]. Furthermore, IL-1β found at high levels in the hippocampus [55] could be involved in cognitive deficits [56]. Our results reveal that after *Pb*A-infection, the microglial production of IL-1β which affects neurogenesis and causes cognitive impairments, is mediated by IL-33/ST2 pathway, prior to the apparition of ECM-induced clinical neurological symptoms. Furthermore, it has been described *in vitro* that IL-1β stimulation impacts oligodendrocytes [43]. Considered originally as passive cells, oligodendrocytes have also the capacity to express several immunomodulatory molecules such as IL-33, as well as many receptors, as shown in EAE model [57]. Furthermore, after CNS injury, damaged oligodendrocytes immediately release IL-33, which acts on local astrocytes and microglia to induce the discharge of chemokines, leading to monocytes recruitment to the brain [23]. Oligodendrocyte derived IL-33 contributed to experimental neuropathic pain [24]. Here, we show a prominent production of IL-33 by essentially all oligodendrocytes (98%) in the hippocampus after *Pb*A-infection, which does not formally exclude a contribution of the other IL-33 producing cells such as astrocytes, and this was dependent on the IL-33/ST2 pathway.

In glial cell cultures, we revealed that IL-33, through ST2 pathway, is sufficient to stimulate microglial cells to produce IL-1β. Conversely, IL-1β induced oligodendrocytes to express IL-33. Based on our *in vivo* and *in vitro* results, we propose a CNS amplification loop between IL-1β and IL-33 produced by microglia and oligodendrocyte respectively, which may contribute to the early cognitive defects before ECM development.

The role of IL-1β in ECM and associated neurological defects is still poorly defined. Indeed, although administration of low doses of IL-1 was originally found to protect mice against cerebral malaria, through T-cells and IFNγ [58], postmortem analysis detected increased IL-1β in human brains with cerebral malaria [59]. While mice deficient for IL-1R or inflammasome developed ECM after blood stage *Pb*A-infection [60], MyD88-deficient mice were resistant to ECM after sporozoite *Pb*A-infection, less so after blood-stage infection, pointing to a role of IL-1R1 pathway on the pre-hepatic stage of infection. However, mice deficient for IL-1β, IL-18 or caspase 1 were as susceptible as wild-type mice to both blood stage and sporozoite *Pb*A-infection [61]. Here we further hypothesized a role for IL-1 pathway not only in the immune response leading to ECM development but also on the CNS inflammatory response and potential cognitive associated defects.

Myelination, a crucial process for signal transmission and neuronal survival, is affected by microglia inflammatory cytokine production in various diseases. Microglia derived-IL-1β was recently reported to induce hypomyelination by suppressing the maturation of oligodendrocyte progenitor cells [43]. Here, oligodendrocyte and myelination markers *Olig2* and *Mbp* transcripts were not different in *Pb*A-infected WT and ST2 deficient mice. In line with our results, oligodendrocytes were not affected but the structure of myelin *per se* was impacted in *Pb*A-infected WT mice [62]. Furthermore, IL-33 may play a role of inhibitor of myelination in multiple sclerosis model [63]. Thus, IL-1β produced by microglia in response to IL-33 induced a sharp increase of IL-33 expression by oligodendrocytes and this important IL-33 release in hippocampus may cause myelin structure defects.

**Fig 9:**
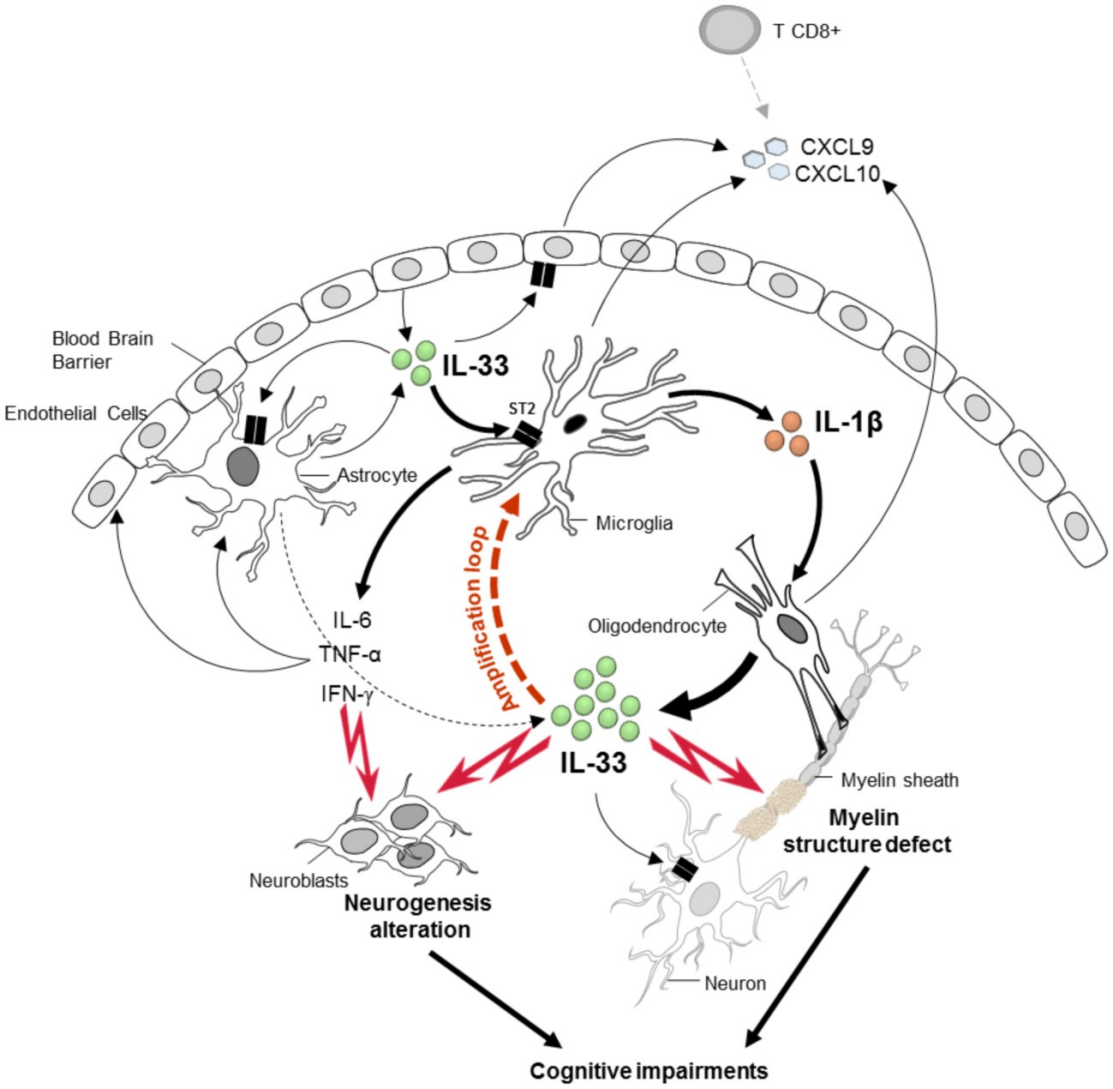
Crucial role of IL-33/ST2 pathway in cognitive and neurological impairments after *Pb*A-infection. In the CNS IL-33 is expressed by endothelial cells, astrocytes and prominently by oligodendrocytes. After *Pb*A-infection, IL-33 induces IL-1β release by microglia, contributing to the high levels of IL-1β, TNF-α and IFN-γ production seen at an early stage of infection. The neuroinflammatory response induced by IL-33/ST2 pathway alters neuroblast proliferation, associated with neurogenesis impairment and cognitive defects. In addition, IL-1-β induced by microglia, in turns stimulates oligodendrocytes to produce high levels of IL-33, leading to an amplification loop. IL-33 overexpression by oligodendrocytes induced an inflammatory context which could affect the myelin structure, as reported after *Pb*A-infection in WT mice [62], and this could be responsible for some cognitive impairments. Furthermore, IL-33/ST2 pathway orchestrates CNS glial cell response at an early stage of *Pb*A-infection, promoting CXCL9 and CXCL10 production to recruit, in a second phase the ECM pathogenic CD8^+^ T cells in cerebral vessels.

In conclusion (Fig 9), we demonstrated that IL-33/ST2 pathway contributes early and directly to the exacerbated neuroinflammation during ECM development with microglial inflammatory cytokine release, affecting the neurogenesis and cognitive processes, in addition to promoting pathogenic CD8^+^ T cells recruitment in brain vessels [11]. Furthermore, IL-33/ST2 pathway induced microglia to produce IL-1β that in turn may cause oligodendrocyte stimulation and IL-33 production, in an amplification loop that may affect myelination and cognition. This study provides new mechanistic insights into the development of cognitive impairments induced after *Plasmodium* infection, and may contribute to the understanding of cerebral malaria neuropathology.

## Materials and methods

### Ethics statement

All animal experimental protocols complied with the French ethical and animal experiments regulations (see Charte Nationale, Code Rural R 214–122, 214–124 and European Union Directive 86/609/EEC) and were approved by the “Ethics Committee for Animal Experimentation of CNRS Campus Orleans” (CCO), registered (N°3) by the French National Committee of Ethical Reflexion for Animal Experimentation, under N° CLE CCO 2012–050.

### Mice

IL-33/citrine (cit) reporter mice (IL-33cit/+) [26] and mice deficient for ST2 receptor (ST2^-/-^) [64] were obtained from Dr. Andrew McKenzie (Laboratory of Molecular Biology, Cambridge University, Cambridge, United Kingdom). ST2^-/-^ were obtained 10-fold back-crossed on C57BL/6J and genetic background monitored by microsatellite analysis (S8 Fig). All mice were bred in the Transgenose Institute animal facility (UPS44 CNRS, Orleans) and wild-type C57BL/6J mice were used as control.

### Experimental malaria infection

*Plasmodium berghei* ANKA (*PbA*) 15cy1 line constitutively expressing GFP under EF1α-promoter control was obtained from Dr. A. Waters [65]. Mice were infected intraperitoneally with 10^5^ parasitized erythrocytes as described [66]. Mice were observed daily and scored for ECM neurological signs, namely ruffled fur, hunching, wobbly gait, limb paralysis, coma or death (from 1 to 5), a clinical score superior to 3 was considered ECM-related. BBB integrity was determined using Evans Blue (EB) dye extravasation into brain tissue as reported previously [36, 67].

### Parasitemia

Parasitaemia was analyzed with EGFP-*Pb*A as described [66] and fluorescent cells assessed by a BD CANTO II flow cytometer. Data were acquired by using DIVA software (BD Bioscience, Rungis, France) and analyzed with FlowJO software (Treestar, Ashland, USA).

### Short-term memory test

*The new object recognition* (NOR) test was performed on day 5 post *Pb*A-infection (5 dpi). Control and infected mice were acclimatized to a novel arena (open field 40 cm x 40 cm) for 5 min. Twenty-four hrs after, a training session was conducted with two identical objects positioned in two opposite corners (at 8 cm from the walls) of the arena for 5 min. The short-term retention memory (STM) test took place 5 min after the training session. During the test session, STM was tested by replacing one of the familiar objects by a novel object and mice explored the open field for 5 min. New (N) and familiar (F) objects were distinct in shape and color. To rule out position bias in exploration, the position of the novel object relative to the familiar object was changed across animals in a randomized fashion. The distance traveled and time spent investigating the objects were measured using Ethovision software (version 10, Noldus Technology, The Netherlands). The exploratory preference was defined according to Bevins and Besheer (2006) as the percentage of the total time that animal spent investigating the new object. It was calculated by the ratio TN/(TF+TN) * 100 (where TN and TF = time spent exploring the new and familiar objects, respectively).

### Y maze spontaneous alternation test

The Y maze apparatus consisted of three identical arms spaced at 120° from each other and located in a room containing different extra-maze cues. The Y maze was performed to test for spatial memory and included two phases. During a training test, mice were placed into one of the arms (start arm) for 5 min and explored the maze with one arm opened (familiar arm) and one arm closed (new arm). After 10 min rest in a separate cage, the mouse was reintroduced in the start arm for the test session and allowed to freely explore the entire maze including the new arm for 2 min. To avoid bias, each arm was alternatively used as start arm. The time spent in each arm, familiar vs new arm, was measured from the videos using the Ethovision software (version 10, Noldus Technology, The Netherlands). The spatial memory was defined as the mouse capacity to recognize the new arm. It was calculated by the ratio TN/(TF+TN)*100, where TN and TF = time spent exploring the new and familiar arms, respectively.

### Tissue preparation and immunofluorescence

Animals were terminally anesthetized with a ketamine (100 mg/mL) / xylazine (20 mg/mL) combination and brain was harvested from the animals after gravity perfusion with heparinized (2mM) PBS followed by 4% paraformaldehyde (PFA). Then, tissue was post-fixed for 48 hours in 4% PFA, followed by cryoprotection in 20% sucrose in PBS for 3 weeks. Brain tissue was imbedded in OCT media (Tissue Tek, Sakura), and 12 μm sections were cut in a cryostat and stored at -20°C until immunofluorescence. The slides were incubated in citrate buffer (citric acid 1M, sodium citrate) at 80°C for 30 min, followed by 3 washes in TBS. They were then blocked during 45 min in a wet chamber at RT in blocking buffer (TBS 1X; 1% BSA; 10% FCS; 0.3% Triton; 1% NaN3) followed by incubation with primary antibody at 4°C overnight. Immunofluorescence staining was performed with antibodies raised in rabbit anti-GFAP (Invitrogen 18–0063; 1:500), rabbit anti-OLIG2 (Millipore AB9610; 1:500), mouse anti-MAP2 (Sigma M9942; 1:500), goat anti-IBA1 (Abcam ab5076; 1:500), rabbit anti-DCX (Abcam ab77450; 1:500), rabbit anti-Ki67 (Abcam ab15580; 1:500), goat anti-IL-33 (R&D Systems AF3626; 1:100). After 3 washes in TBS, the corresponding conjugated secondary antibodies were incubated for 2 hrs at RT. Slides were then rinsed in TBS, counter-stained with DAPI for 10 min, mounted with Fluoromount-G (SouthernBiotech), and dried before observation using a Leica microscope (DM6000B, Leica microsystems, Heidelberg, Germany) powered by Metamorph software. Image analysis and processing were performed with the public domain software ImageJ (NIH).

### Brain imaging using MRI and MRA

MRI and MRA measurement of cerebral vascular blood flow were performed on a 7T horizontal superconducting magnet dedicated to small animal imaging (70/16 USR Bruker Pharmascan, Wissembourg, France) and equipped with a 330mT/m gradient set. A Bruker 28 mm inner diameter birdcage coil was used for both 1H transmission and reception. During the MR experiments, mice were positioned in a custom-built cradle to immobilize the head. They were anesthetized with isoflurane (1.5%) and a mixture of O2/N2O (1:1) with an output of 0.5 L/min. Respiration was monitored during the whole experiment using an air pillow and body temperature was maintained constant at 37°C by a warm-water circulation system-MRI.

T1 weighed images were performed before and after administration of Gadolinium (Gd) solution (150μL 2mmol/kg DOTAREM, Guerbet, France) via the tail caudal vein. The sequence parameters were: TE/TR = 5 ms/16 ms, FOV = 20 x20 mm2, matrix = 256 x 256, slice thickness = 0.5 mm, experimental time = 2 min. Levels of grey were assessed on original IRM data and representative images are shown with identically increased contrast to point to the affected areas (arrow). Measurements of vascular cerebral blood flow was performed by MRA using a Fast Low Angle Shot (FLASH) sequence, with the following parameters: FOV = 18 x18 mm2, matrix = 256 x 256, TR/TE = 16/5 ms, slice thickness = 0.2 mm, experimental time = 11 min.

### Quantitative real-time PCR

RNA was extracted from tissues as described [68]. RNA was isolated from two brain regions, hippocampus and frontal cortex using TRI-Reagent (Sigma). Reverse transcription was performed with SuperScript III Kit (Invitrogen) and cDNA was subjected to quantitative real-time PCR using primers for *Ifng*, *Tnfa*, *Il6*, *Il1b*, *Il1r1*, *Cxcl10*, *Cxcl9*, *Cd8a*, *Olig2* and *Mbp* (Qiagen) and GoTaq qPCR-Master Mix (Promega). Raw data were normalized to *18s* expression and were analyzed by the 2^ΔΔCt^ method [69].

### Primary glial cell cultures

Primary glial cell cultures containing astrocytes, microglia and oligodendrocytes were prepared from mouse neonates (post-natal day 0–4). After brain dissection, the meningeal layer was removed and the cortex isolated and incubated in a solution containing papain (1.2μg/ml), DNase I (0.02%), and cysteine in glial culture media (Dulbecco's modified Eagle's medium (DMEM) supplemented with 10% fetal calf serum (FCS), 1% penicillin and streptomycin, 25mM L-glutamine) at 37°C for 5 min. After addition of a stop solution (1mg/ml Trypsin inhibitor, 0.02% DNase in L15 medium), the tissue preparation was resuspended and centrifuged at 100g for 2 min. The pelleted cells were slowly resuspended in glial culture medium and incubated in a poly-L-Lysine (PLL) coated T25 flask under 5% CO_2_ at 37°C. Twenty-four hrs later, the medium was changed and thereafter every 3–5 days. The cells were confluent after 10 days in culture. Before trypsin treatment, the cells were shaked at 250 RPM overnight at room temperature and the medium removed. The harvested cells were then sub cultured once and seeded (2.10^5^ cells/well) on PLL coated glass coverslips placed in 24 well plates and incubated in 5% CO_2_ at 37°C. After 24 hrs, the medium was refreshed and changed every 3–5 days thereafter. Under these *in vitro* conditions, the cells obtained on day 15 were ca 85% astrocytes, 15% oligodendrocytes and 10% microglia.

### Treatment of primary glial cell cultures

Primary glial cell cultures on coverslips were stimulated with LPS (InvivoGen tlrl-3pelps; at 10μg/mL for 24 hrs) as a positive control for IL-33 and IL-1β expression. Alternatively, cultures were incubated with recombinant mouse IL-1β (R&D Systems 401-ML/CF; at 30ng/mL) or recombinant mouse IL-33 (R&D Systems 3626-ML; at 10 and 100ng/mL) for 24 hrs. Glial cells were washed three times for 5 min in PBS, then fixed 10 min with 4% PFA, and further washed 3 times in PBS. Microscope coverslips were then stored at 4°C for immunostaining analysis.

### Immunostaining of primary glial cells

Cells were washed with TBS and blocked in solution containing 10% FCS, 0.2% Triton in TBS and 1% BSA for 45 min at RT. The cells were incubated overnight with different primary antibodies raised in rat anti-CD11b (Bio-Rad MCA711G; 1:200), goat anti-IL-1β (Sigma I3767; 1:500), or rabbit anti-GFAP, rabbit anti-OLIG2, goat anti-IL-33 as above. After 3 washes in TBS, the corresponding conjugated secondary antibodies were incubated for 45 min at RT in a solution containing 10% FCS and 1% BSA in TBS. Cells were rinsed in TBS and counter-stained with DAPI for 1 min at RT. After 3 final washes with TBS, the coverslips were mounted on glass slides with Fluoromount-G and image analysis performed as above.

### Statistical analysis

Statistical significance was determined with GraphPad Prism v6 (GraphPad Software, La Jolla, CA). To analyze non-parametric data, Mann-Whitney test for 2 series was used or Kruskal-Wallis followed by Dunn’s multiple comparison for more sample. One-way ANOVA followed by Bonferroni post-test was performed to evaluate difference between 4 or more series, with parametric data. Logrank test for survival was applied. *P* values ≤0.05 were considered statistically significant.

## Supporting information

S1 Fig IL-33 regulation after *Pb*A-infection in the subventricular zone and the cerebellum. (A-B) Brain sections from naïve or 7-day-*Pb*A-infected IL-33/citrine reporter mice were analyzed to get representative images and cell counts from subventricular zone (A) and cerebellum (B), with fluorescent citrine to visualize *Il33* transcript and with immunofluorescence staining of IL-33 protein with an antibody anti-IL-33. Scale bar 100μm. These results are representative of 2 independent experiments and shown as mean ± SEM with n = 5. Mann Whitney test was applied (*p≤0.*05*, ***p≤0.*001)*.
(TIF)

S2 Fig *Pb*A-induced impairment of cognition is prevented in the absence of IL-33/ST2 pathway. (A-B) Locomotor activity measured during both behavioral tests for WT and ST2^-/-^ mice at day 5 post-*Pb*A infection compared to naïve mice. These results are shown as mean ± SEM with n = 10 to 15 mice for WT and n = 9 to 10 for ST2^-/-^ mice. Kruskal-Wallis was applied, followed by Dunn’s comparison test.
(TIF)

S3 Fig Preserved cognition 11 days post-*Pb*A infection in the absence of IL-33/ST2 pathway. (A) Y maze was used to assess hippocampal memory in ST2 deficient mice, either naïve (D0) or on day 11 post-*Pb*A infection (D11). (B) Locomotor activity measured during Y maze test. The results are shown as mean ± SEM with n = 9 to 5 mice, respectively. Mann Whitney test was applied (**p≤0.*01)*.
(TIF)

S4 Fig Microglia activation in cerebellum associated with ECM, independently of IL-33/ST2 pathway. (A) Immunofluorescent staining of microglia marker IBA1, on brain sections, showing microglia morphology in cerebellum from WT and ST2^-/-^ mice, 7 days post-*Pb*A-infection. Scale bar 20μm. (B) Cell counts of IBA1^+^ cells from A, to compare microglia proliferation in WT and ST2^-/-^ brain, 7 days post-*Pb*A infection. These results are expressed as mean ± SEM, n = 3 per group in 2 independent experiments. Kruskal-Wallis was applied, followed by Dunn’s comparison test (*p≤0.*05)*.
(TIF)

S5 Fig Inflammatory response in frontal cortex after *Pb*A-infection is reduced in the absence of IL-33/ST2 pathway. Inflammation context in frontal context was investigated in naïve or *Pb*A-infected WT or ST2^-/-^ mice on day 5, 6 and 7 after infection. (A-D) *Ifng*, *Tnfa*, *Il1b* and *Cd8a* mRNA expressions in frontal cortex were quantified by real-time quantitative RT-PCR. Expression of *18s* housekeeping gene was used for normalization. Results expressed as fold change relative to uninfected mice are mean ± SEM, n = 5 mice per group per day, representative of 3 independent experiments. Statistical analysis was done using Kruskal-Wallis test followed by Dunn’s comparison test (*p≤0.*05)*.
(TIF)

S6 Fig Expression of IL-33 and IL-1β by primary glial cell cultures. WT mixed glial cell cultures from newborn mice were stimulated with LPS (10μg/mL), as a positive control, for 24 hrs then fixed and immunostained to determine the cellular source of IL-33 and IL-1β. (A-B) Immunofluorescence staining of IL-33 (A) and IL-1β (B) with astrocyte marker GFAP, oligodendrocyte marker OLIG2 and microglia marker CD11b. These images are representative of 3 independent experiments. Arrows indicate colocalization. Scale bar 100μm.
(TIF)

S7 Fig IL-1β protein expression in hippocampus after *Pb*A-infection. Hippocampus expression of IL-1β protein was analyzed by ELISA, on day 5, 6 and 7 post-*Pb*A infection in WT and ST2 deficient mice compared to naïve mice. The results are expressed as mean ± SEM, with n = 5 per day.
(TIF)

S8 Fig Microsatellite genetic analysis of ST2^-/-^ mice. C57BL/6, 129Sv and ST2^-/-^ mice [49] were analyzed by PCR with a series of microsatellite markers to verify B6 genetic background for each chromosome of ST2^-/-^ mice. Representative PCR gels are shown for chromosome D1Mit303 (A), D2Mit395 (B), D4Mit193 (C), D5Mit161 (D), D6Mit36 (E), D13Mit191 (F), D14Mit60 (G) and DXMit136 (H). The whole series of microsatellites tested comprised D1MIT3, D1Nds9, D1Mit303, D2mit148, D2Mit395, D3Mit203, D4Mit193, D4Mit308, D5MIT24, D5MIT188, D5Mit73, D5Mit161, D6Mit8, D6Mit36, D7MIT122, D7Mit158, D7Mit220, D8Mit178, D9Mit250, D10MIT10, D10Mit108, D10Mit230, D11MIT20, D11MIT5, D11Mit349, D11Mit333, D12Mit12, D12Mit99, D13Mit16, D13Mit191, D14Mit60, D14Mit95, D15Mit13, D15Mit154, D16MIT4, D16Mit140, D17Mit164, D17Mit205, D18Mit177, D19MIT1 and DXMit136.
(TIF)
