# Supplementary figures and images for: IL-33 receptor ST2 regulates the cognitive impairments associated with experimental cerebral malaria
Source: PLoS Pathog. 2017 Apr 27;13(4):e1006322. doi: 10.1371/journal.ppat.1006322 (PMC5407765; doi:10.1371/journal.ppat.1006322)

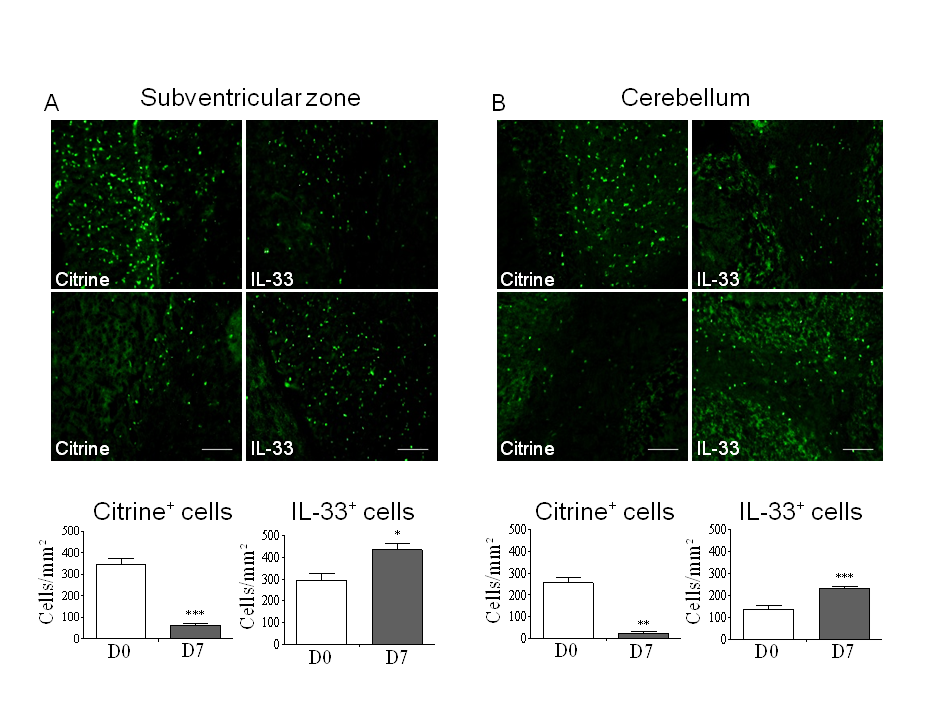

Supplement: S1 Fig — (A-B) Brain sections from naïve or 7-day-PbA-infected IL-33/citrine reporter mice were analyzed to get representative images and cell counts from subventricular zone (A) and cerebellum (B), with fluorescent citrine to visualize Il33 transcript and with immunofluorescence staining of IL-33 protein with an antibody anti-IL-33. Scale bar 100μm. These results are representative of 2 independent experiments and shown as mean ± SEM with n = 5. Mann Whitney test was applied (*p≤0.05, ***p≤0.001). (TIF) [file ppat.1006322.s001.tif]

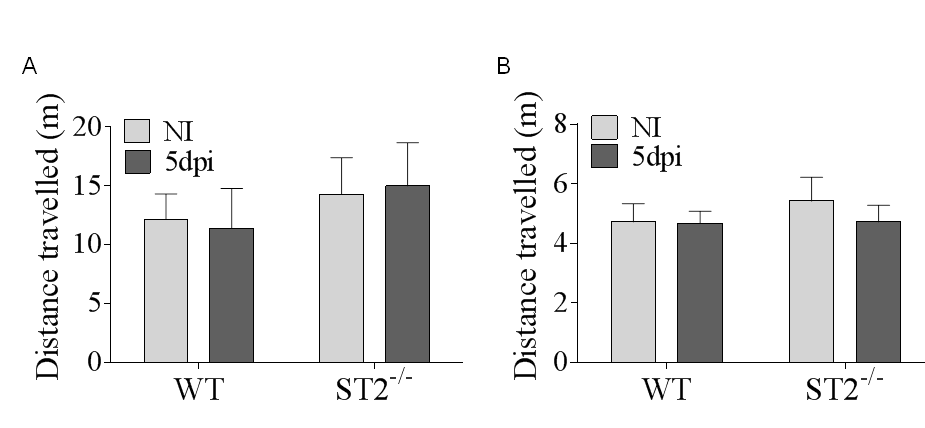

Supplement: S2 Fig — (A-B) Locomotor activity measured during both behavioral tests for WT and ST2-/- mice at day 5 post-PbA infection compared to naïve mice. These results are shown as mean ± SEM with n = 10 to 15 mice for WT and n = 9 to 10 for ST2-/- mice. Kruskal-Wallis was applied, followed by Dunn’s comparison test. (TIF) [file ppat.1006322.s002.tif]

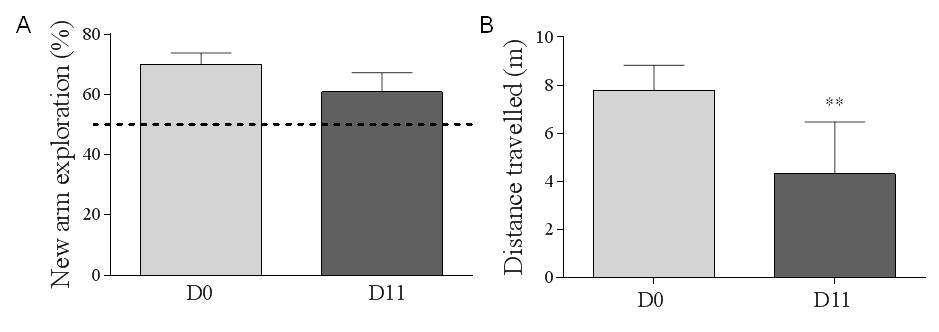

Supplement: S3 Fig — (A) Y maze was used to assess hippocampal memory in ST2 deficient mice, either naïve (D0) or on day 11 post-PbA infection (D11). (B) Locomotor activity measured during Y maze test. The results are shown as mean ± SEM with n = 9 to 5 mice, respectively. Mann Whitney test was applied (**p≤0.01). (TIF) [file ppat.1006322.s003.tif]

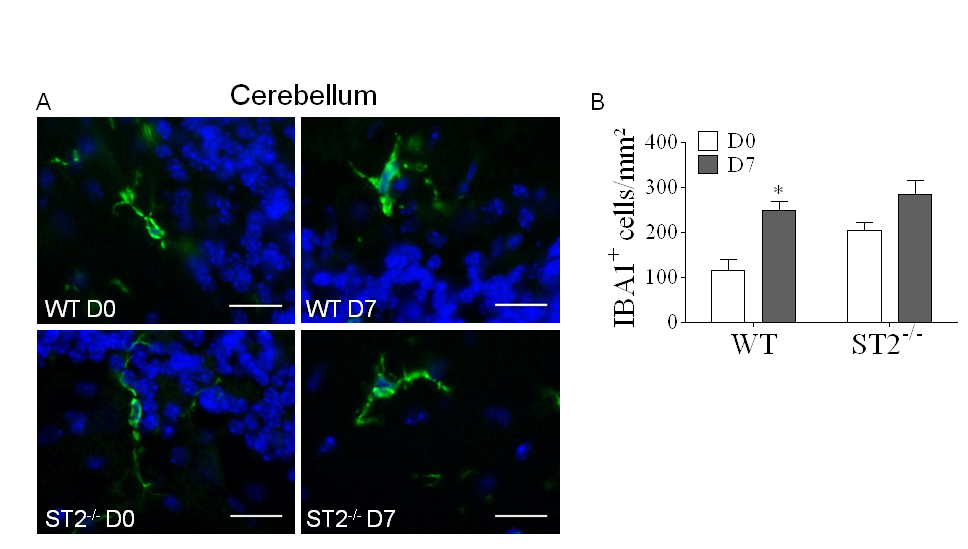

Supplement: S4 Fig — (A) Immunofluorescent staining of microglia marker IBA1, on brain sections, showing microglia morphology in cerebellum from WT and ST2-/- mice, 7 days post-PbA-infection. Scale bar 20μm. (B) Cell counts of IBA1+ cells from A, to compare microglia proliferation in WT and ST2-/- brain, 7 days post-PbA infection. These results are expressed as mean ± SEM, n = 3 per group in 2 independent experiments. Kruskal-Wallis was applied, followed by Dunn’s comparison test (*p≤0.05). (TIF) [file ppat.1006322.s004.tif]

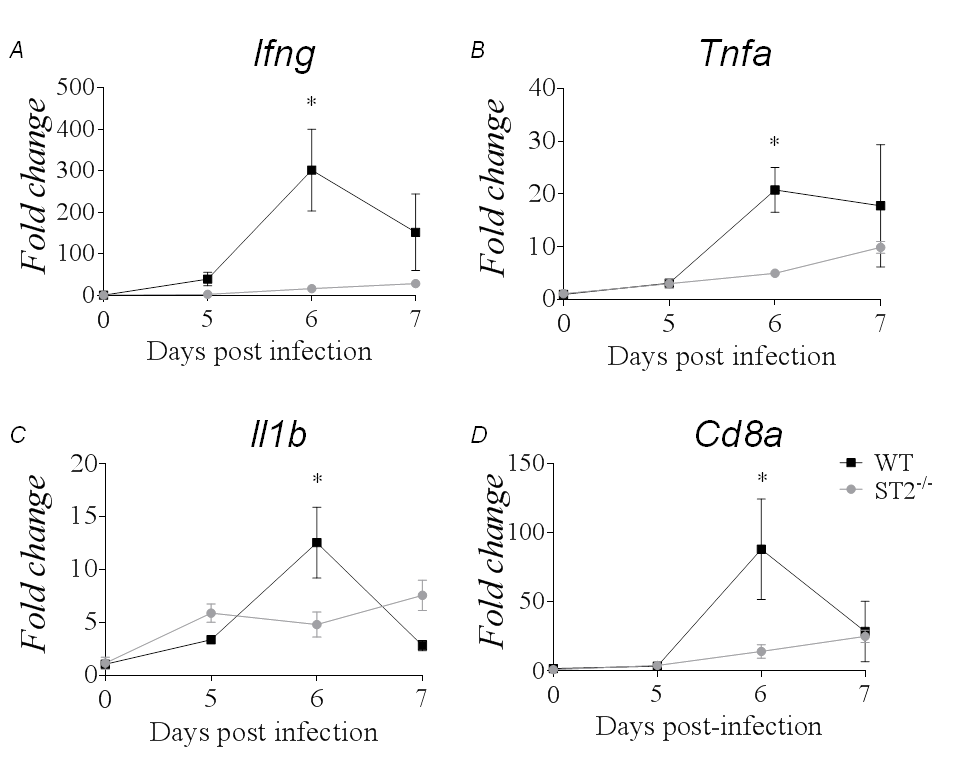

Supplement: S5 Fig — Inflammation context in frontal context was investigated in naïve or PbA-infected WT or ST2-/- mice on day 5, 6 and 7 after infection. (A-D) Ifng, Tnfa, Il1b and Cd8a mRNA expressions in frontal cortex were quantified by real-time quantitative RT-PCR. Expression of 18s housekeeping gene was used for normalization. Results expressed as fold change relative to uninfected mice are mean ± SEM, n = 5 mice per group per day, representative of 3 independent experiments. Statistical analysis was done using Kruskal-Wallis test followed by Dunn’s comparison test (*p≤0.05). (TIF) [file ppat.1006322.s005.tif]

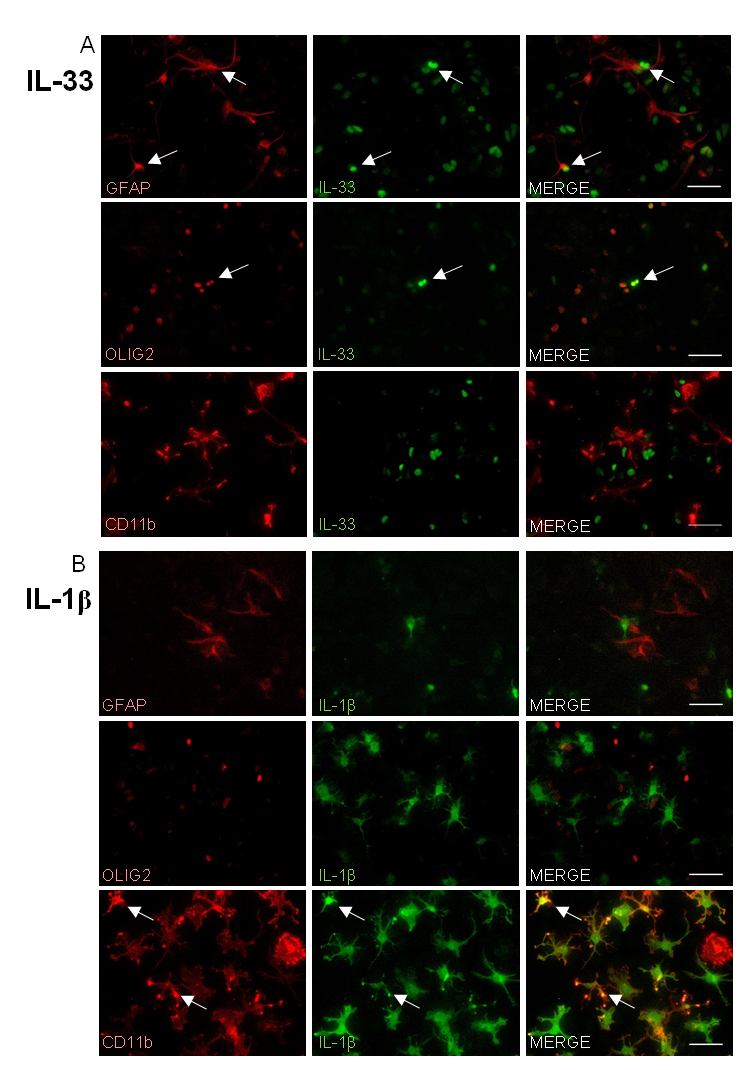

Supplement: S6 Fig — WT mixed glial cell cultures from newborn mice were stimulated with LPS (10μg/mL), as a positive control, for 24 hrs then fixed and immunostained to determine the cellular source of IL-33 and IL-1β. (A-B) Immunofluorescence staining of IL-33 (A) and IL-1β (B) with astrocyte marker GFAP, oligodendrocyte marker OLIG2 and microglia marker CD11b. These images are representative of 3 independent experiments. Arrows indicate colocalization. Scale bar 100μm. (TIF) [file ppat.1006322.s006.tif]

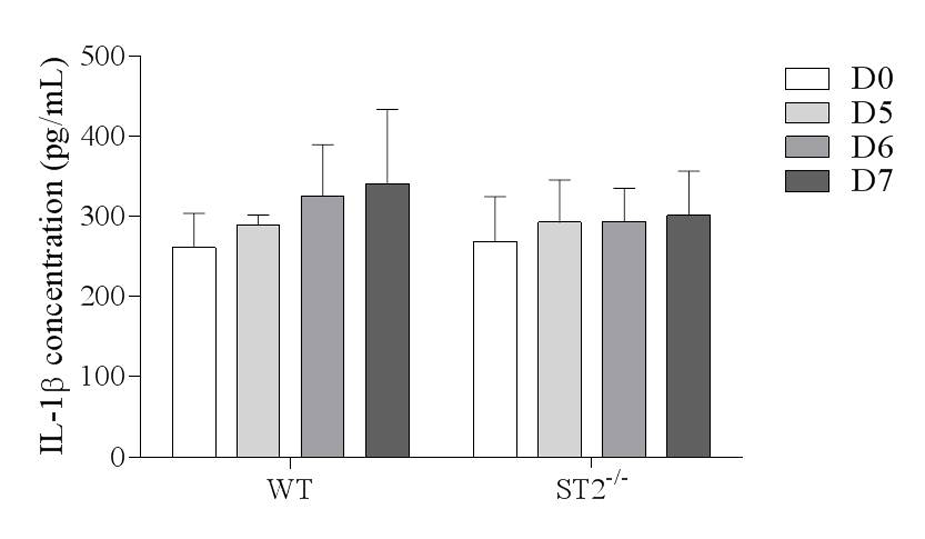

Supplement: S7 Fig — Hippocampus expression of IL-1β protein was analyzed by ELISA, on day 5, 6 and 7 post-PbA infection in WT and ST2 deficient mice compared to naïve mice. The results are expressed as mean ± SEM, with n = 5 per day. (TIF) [file ppat.1006322.s007.tif]

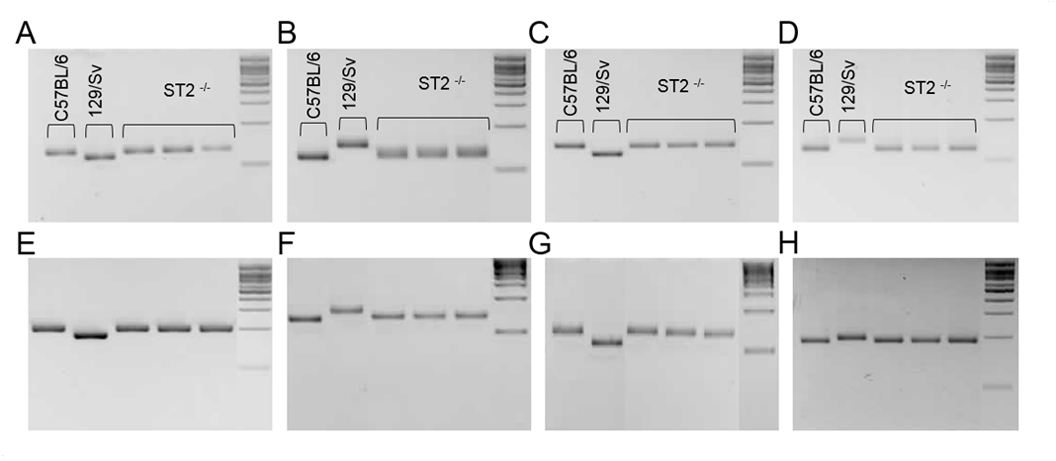

Supplement: S8 Fig — C57BL/6, 129Sv and ST2-/- mice [49] were analyzed by PCR with a series of microsatellite markers to verify B6 genetic background for each chromosome of ST2-/- mice. Representative PCR gels are shown for chromosome D1Mit303 (A), D2Mit395 (B), D4Mit193 (C), D5Mit161 (D), D6Mit36 (E), D13Mit191 (F), D14Mit60 (G) and DXMit136 (H). The whole series of microsatellites tested comprised D1MIT3, D1Nds9, D1Mit303, D2mit148, D2Mit395, D3Mit203, D4Mit193, D4Mit308, D5MIT24, D5MIT188, D5Mit73, D5Mit161, D6Mit8, D6Mit36, D7MIT122, D7Mit158, D7Mit220, D8Mit178, D9Mit250, D10MIT10, D10Mit108, D10Mit230, D11MIT20, D11MIT5, D11Mit349, D11Mit333, D12Mit12, D12Mit99, D13Mit16, D13Mit191, D14Mit60, D14Mit95, D15Mit13, D15Mit154, D16MIT4, D16Mit140, D17Mit164, D17Mit205, D18Mit177, D19MIT1 and DXMit136. (TIF) [file ppat.1006322.s008.tif]
